# Risk Factor Control and Cardiovascular Event Risk in People With Type 2 Diabetes in Primary and Secondary Prevention Settings

**DOI:** 10.1161/CIRCULATIONAHA.120.046783

**Published:** 2020-11-17

**Authors:** Alison K. Wright, Milton Fabian Suarez-Ortegon, Stephanie H. Read, Evangelos Kontopantelis, Iain Buchan, Richard Emsley, Naveed Sattar, Darren M. Ashcroft, Sarah H. Wild, Martin K. Rutter

**Affiliations:** 1 Division of Diabetes, Endocrinology and Gastroenterology, School of Medical Sciences, University of Manchester, United Kingdom (A.K.W., M.K.R.).; 2 Centre for Pharmacoepidemiology and Drug Safety, Division of Pharmacy and Optometry, School of Health Sciences, University of Manchester, United Kingdom (A.K.W., D.M.A.).; 3 Departamento de Alimentación y Nutrición, Facultad de Ciencias de la Salud, Pontificia Universidad Javeriana Seccional Cali, Colombia (M.F.S.-O.).; 4 Grupo de Investigación en Ciencias Básicas y Clínicas de la Salud, Facultad de Ciencias de la Salud, Pontificia Universidad Javeriana Seccional Cali, Colombia (M.F.S.-O.).; 5 Usher Institute of Population Health Sciences and Informatics, University of Edinburgh, United Kingdom (S.H.R., S.H.W.).; 6 Women’s College Research Institute, Women’s College Hospital, Toronto, Ontario, Canada (S.H.R.).; 7 Division of Informatics, Imaging and Data Sciences, School of Health Sciences, University of Manchester, United Kingdom (E.K.).; 8 Department of Public Health and Policy, Institute of Population Health Sciences, University of Liverpool, United Kingdom (I.B.).; 9 Health eResearch Center, Farr Institute, Division of Informatics, Imaging & Data Sciences, School of Health Sciences, University of Manchester, United Kingdom (I.B.).; 10 Department of Biostatistics & Health Informatics, Institute of Psychiatry, Psychology and Neuroscience, King’s College London, United Kingdom (R.E.).; 11 Institute of Cardiovascular & Medical Sciences, University of Glasgow, United Kingdom (N.S.).; 12 Scottish Diabetes Research Network epidemiology group, Scotland, United Kingdom (S.H.W.).; 13 Diabetes, Endocrinology and Metabolism Centre, Manchester University NHS Foundation Trust, Manchester Academic Health Sciences Centre, United Kingdom (M.K.R.).

**Keywords:** cardiovascular risk factors, primary care, primary prevention, risk assessment, secondary care, secondary prevention, type 2 diabetes

## Abstract

To examine the association between the degree of risk factor control and cardiovascular disease (CVD) risk in type 2 diabetes and to assess if the presence of cardio-renal disease modifies these relationships.

**Methods:**

A retrospective cohort study using data from English practices from CPRD GOLD (Clinical Practice Research Datalink) and the SCI-Diabetes dataset (Scottish Care Information-Diabetes), with linkage to hospital and mortality data. We identified 101 749 with type 2 diabetes (T2D) in CPRD matched with 378 938 controls without diabetes and 330 892 with type 2 diabetes in SCI-Diabetes between 2006 and 2015. The main exposure was number of optimized risk factors: nonsmoker, total cholesterol ≤4 mmol/L, triglycerides ≤1.7 mmol/L, glycated haemoglobin (HbA1c) ≤53 mmol/mol (≤7.0%), systolic blood pressure <140mm Hg, or <130 mm Hg if high risk. Cox models were used to assess cardiovascular risk associated with levels of risk factor control.

**Results:**

In CPRD, the mean baseline age in T2D was 63 years and 28% had cardio-renal disease (SCI-Diabetes: 62 years; 35% cardio-renal disease). Over 3 years follow-up (SCI-Diabetes: 6 years), CVD events occurred among 27 900 (27%) CPRD-T2D, 101 362 (31%) SCI-Diabetes-T2D, and 75 520 (19%) CPRD-controls. In CPRD, compared with controls, T2D participants with optimal risk factor control (all risk factors controlled) had a higher risk of CVD events (adjusted hazard ratio, 1.21; 95% confidence interval, 1.12–1.29). In T2D participants from CPRD and SCI-Diabetes, pooled hazard ratios for CVD associated with 5 risk factors being elevated versus optimal risk factor control were 1.09 (95% confidence interval, 1.01–1.17) in people with cardio-renal disease but 1.96 (95% confidence interval, 1.82–2.12) in people without cardio-renal disease. People without cardio-renal disease were younger and more likely to have suboptimal risk factor control but had fewer prescriptions for risk factor modifying medications than those with cardio-renal disease.

**Conclusions:**

Optimally managed people with T2D have a 21% higher CVD risk when compared with controls. People with T2D without cardio-renal disease would be predicted to benefit greatly from CVD risk factor intervention.

## Abstract 2

Supplemental Digital Content is available in the text.

**Clinical Perspective**

**What Is New?**

- Even when cardiovascular disease (CVD) risk factors are optimally controlled, people with type 2 diabetes still have a 21% higher risk of CVD compared with people without diabetes.
- In people with type 2 diabetes without cardio-renal disease, there were much stronger relationships between the degree of risk control and risks for CVD events and mortality than in people with type 2 diabetes with cardio-renal disease.
- People with type 2 diabetes who had no cardio-renal disease were younger than people with cardio-renal disease and had fewer prescriptions for CVD prevention medications.

**What Are the Clinical Implications?**

- Overall risk factor management was poor in people with type 2 diabetes. Greater use of guideline-driven care, clinical decision support, drug intervention, and self-management support should be encouraged.
- People with type 2 diabetes and without cardio-renal disease may benefit greatly from CVD risk factor intervention.

Type 2 diabetes (T2D) is a common condition that increases the risk of fatal and nonfatal cardiovascular disease (CVD).^1,2^ The Steno-2 trial suggested that in people with T2D, multiple CVD risk factor intervention might halve the risk of CVD events and mortality and may provide up to 8 years longer life expectancy^3–6^.

In observational data of people with T2D and coronary disease, we showed that individuals with suboptimal risk factor control had a 2-fold higher risk for mortality and CVD events when compared with those with optimal risk factor control.^7^ A Swedish population-based study recently showed similar findings.^8^ In addition, it showed: 1) that people with T2D who had optimal risk factor control had little or no excess risk of death, myocardial infarction, or stroke when compared with the general population; and 2) that relationships between the degree of risk factor control and CVD events were stronger in younger people than older people, possibly due to differing baseline CVD risks and risk factor management.^7^

Currently, we don’t know whether people in the United Kingdom with T2D who have optimal risk factor control have similar risks of CVD and mortality to people without diabetes, and whether baseline CVD risk modifies the relationship between the degree of risk factor control and CVD risk. This is important because quantifying these risks in individuals considered to be at low-risk or high-risk for CVD could guide appropriate interventions on CVD risk factors. Therefore, in people with T2D, stratified by baseline CVD risk, defined by the presence of cardio-renal disease, we studied associations between the number of abnormal CVD risk factors at baseline with subsequent mortality and CVD events, and we compared CVD risk in optimally controlled people with T2D and controls without diabetes.

## Methods

### Ethical Approval

This study is based in part on data from the CPRD (Clinical Practice Research Datalink) obtained under license from the UK Medicines and Healthcare products regulatory agency. The data are provided by patients and collected by the National Health Service (NHS) as part of their care and support. Office for National Statistics and Hospital Episode Statistics data are subject to Crown copyright (2018) protection, reused with the permission of The Health and Social Care Information Centre, all rights reserved. The Office of Population Censuses and Surveys (OPCS) Classification of Interventions and Procedures, codes, terms, and text is Crown copyright (2016) published by The Health and Social Care Information Centre, also known as NHS Digital and licensed under the Open Government License available at www.nationalarchives.gov.uk/doc/open-government-license/open-government-license.htm. The study and use of CPRD data were approved by the Independent Scientific Advisory Committee for CPRD research (ref. 15_123MnA). Generation of the anonymised, linked SCI-Diabetes dataset (Scottish Care Information-Diabetes) was approved by the Scotland multicenter research ethics committee (reference 11-AL-0225), Caldicott guardians, and the NHS National Services Scotland Privacy Application Committee (reference 33/11). The interpretation and conclusions contained in this study are those of the authors alone.

### Data Sharing

Read and International Classification of Diseases codes used are publicly available at The ClinicalCodes repository and can be accessed at https://clinicalcodes.rss.mhs.man.ac.uk/. The primary care data can be requested via application to the Clinical Practice Research Datalink (https://www.cprd.com); secondary care data can be requested via application to the hospital episode statistics from The Health and Social Care Information Centre (www.hscic.gov.uk/hesdata); and mortality data are available by application to the UK Office for National Statistics (www.ons.gov.uk/ons/index.html). Linked SCI-Diabetes data can be requested via application to the electronic Data Research and Innovation Service (https://www.isdscotland.org/Products-and-Services/eDRIS/).

### Data Sources

This was a retrospective population-based cohort study using data from 2 sources: the Clinical Practice Research Datalink (CPRD GOLD), a UK primary care database (only records from English practices were included in the study due to linkage restrictions), and the Scottish Care Information-Diabetes system (SCI-Diabetes), a Scottish diabetes registry database.

The CPRD is an anonymised, longitudinal primary care medical record database of UK general practices.^9^ In 2015, the CPRD GOLD contained data on over 4.4 million active (alive, currently registered) patients from 674 registered general practices, equating to approximately 6.9% of the UK population.^9^ Patients are broadly representative of the general population in terms of age, sex, and ethnicity.^9^ Approximately 75% of CPRD GOLD practices are located in England (58% of all 674 UK CPRD practices) have consented and participate in the CPRD linkage scheme. The CPRD dataset was linked at the patient-level to Hospital Episode Statistics, Office for National Statistics mortality data, and Index of Multiple Deprivation 2010, for all eligible patients in 380 English practices.

The SCI-Diabetes dataset, a national diabetes system established in 2000, contains data on >99% of all individuals diagnosed with diabetes in Scotland.^10^ SCI-Diabetes is a fully integrated shared electronic patient record to support treatment of National Health Service Scotland patients which includes demographics and primary and secondary care information relevant to diabetes care.^11^ In 2015, SCI-Diabetes contained data on 284 122 people diagnosed with diabetes in Scotland (5.3% prevalence) of which 10.7% were registered with type 1 diabetes and 88.3% with T2D.^12^ The Information Services Division of NHS National Services Scotland linked a 2016 extract of SCI-Diabetes data to national mortality records and hospital data from the Scottish Morbidity Records (SMR01).

### Study Populations

**Figure 1.**
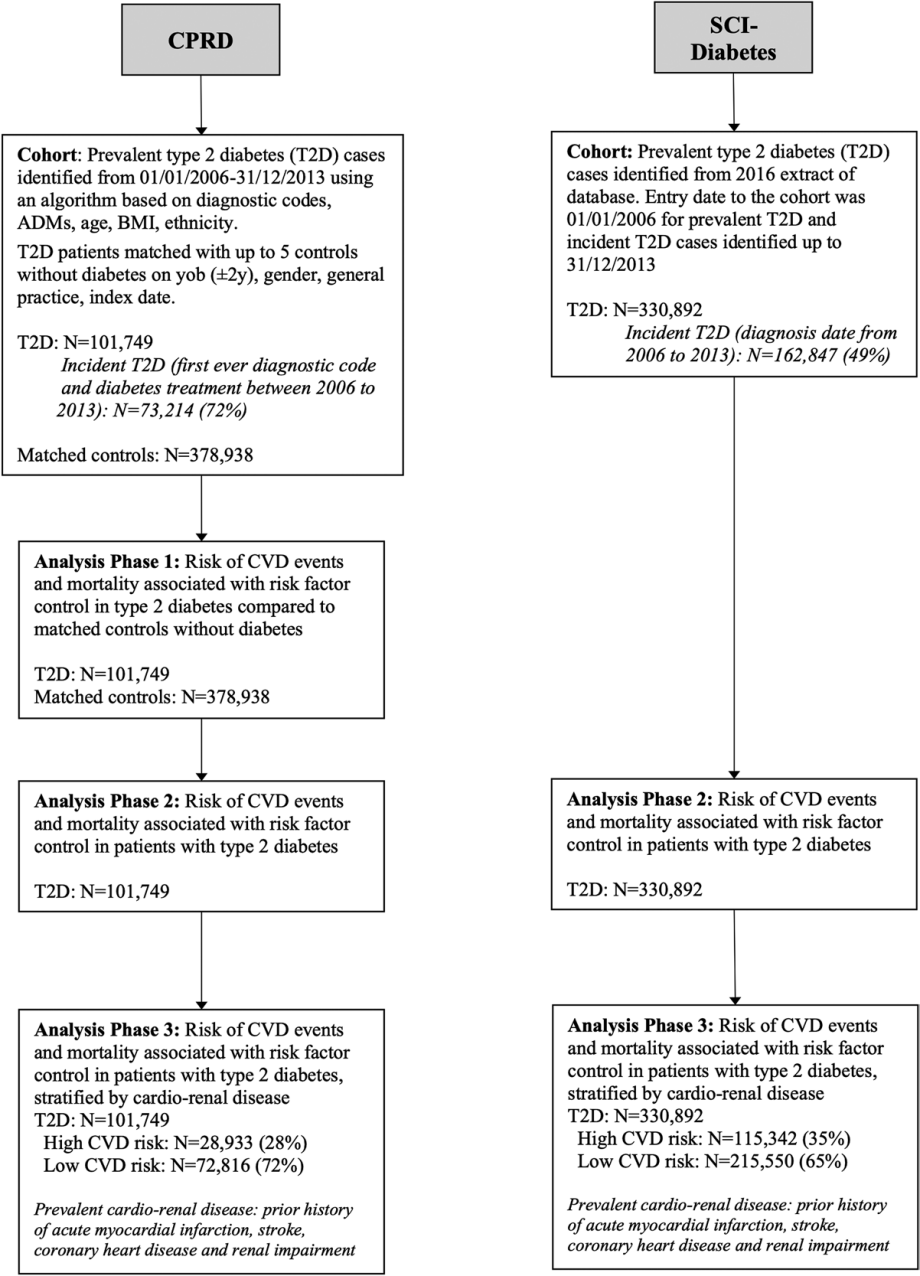
**Study populations from CPRD (Clinical Practice Research Datalink) and SCI-Diabetes (Scottish Care Information-Diabetes) at each analytic phase.** ADMs indicates antidiabetic medications; BMI, body mass index; CVD, cardiovascular disease; and T2D, type 2 diabetes.

Figure 1 outlines the study populations included at each analytic phase.

#### CPRD

T2D cases were identified from Read codes (coded thesaurus of clinical terms used in primary care^13^) in the electronic record between January 1, 2006, and December 31, 2013. A validated algorithm classified people with T2D based on diabetes codes available, diabetes treatments, age, body mass index, and ethnicity as described previously.^1,14^ The index date in the T2D cohort was defined as the first diagnostic code or diabetes treatment within the study window. Incident T2D was defined if there was no history of diagnostic codes for diabetes or diabetes treatments prior to the index date.

People with T2D were matched with up to 5 controls without diabetes (any type) on year of birth (± 2 years), sex, general practice, and index date. For cohort entry, individuals with T2D and controls without diabetes were required to have at least 1 year prior registration at their current practice and for the practice to be up-to-standard for research purposes (a CPRD practice-based quality metric based on continuity and accuracy of data recording). All participants were observed from the index date to the end point date; the study end date (March 31, 2015), the practice’s last data collection date, death, or transfer out of practice, whichever occurred first.

#### SCI-Diabetes

People with T2D were identified from a 2016 extract of the SCI-Diabetes database in which diabetes type is recorded by a clinician at diagnosis. For research purposes, an algorithm based on age at diagnosis, use, and timing of treatment with oral hypoglycaemics and insulin is applied to validate type of diabetes.^15^ The entry date to the cohort was January 1, 2006, for people with prevalent T2D and the date of T2D diagnosis for those diagnosed after that date. Participants were observed from cohort entry to the earliest outcome date, death, or the study end date (March 31, 2015).

### Cardiovascular Outcomes

In both datasets, information on cardiovascular outcomes was identified from hospital records (Hospital Episode Statistics in England and SMR01 in Scotland), detailed in Figure I in the Data Supplement, and death records (Office for National Statistics in England and national death registrations in Scotland) using the following ICD-10 codes: coronary heart disease (I20-I25), stroke (I60-I64), hospitalisation for heart failure (I50), and other forms of heart disease (I30-I52).

Primary study outcome included a composite of total CVD events (fatal/nonfatal coronary heart disease (CHD), stroke, or heart failure hospitalisation). Secondary outcomes included nonfatal CHD, nonfatal stroke, nonfatal heart failure, total CVD mortality, fatal CHD, and fatal stroke.

### Patient Demographics and Baseline Clinical Characteristics

In CPRD, ethnicity was identified from the primary care records using Read codes and through Hospital Episode Statistics linkage as described previously.^1^ In SCI-Diabetes, ethnicity is self-assigned by the person with diabetes using the ethnic groups list from the 2001 Scottish Census.^15^ In CPRD and SCI-Diabetes, ethnicity was categorized into 4 groups: White, South Asian, Black, and Other. Deprivation data were defined using the Index of Multiple Deprivation 2010 classification in CPRD and the Scottish Index of Multiple Deprivation in SCI-Diabetes both categorized into 5 quintiles: 1 (most deprived) to 5 (least deprived).

In CPRD, history of CVD at baseline was defined using Read codes and ICD-10/OPCS-4 (OPCS Classification of Interventions and Procedures version 4; NHS coding for interventions and surgical procedures^16^) codes before the index date for the following cardiovascular conditions: myocardial infarction, stroke, heart failure, CHD, cerebrovascular disease, peripheral vascular disease, and revascularisation interventions. For the SCI-Diabetes cohort, prevalent CVD was defined based on ICD-10/OPCS-4 (Office of Population Censuses and Surveys [OPCS] Classification of Interventions and Procedures version 4) codes alone.

For both CPRD and SCI-Diabetes, moderate to severe renal impairment that is likely to affect treatment decisions was defined using eGFR values <45 mL/min/1.73m^2^. Risk factors (smoking, body mass index, total cholesterol, triglycerides, glycated haemoglobin [HbA1c], and blood pressure) were identified from the closest recording up to 1 year before or after the index date. Drug prescriptions for antidiabetics, antihypertensives, lipid-lowering therapies, and antiplatelet agents were defined in the period up to 3 months prior to index date.

### Definition of Risk Factor Control

In CPRD and SCI-Diabetes, individuals with T2D were categorized into 6 groups, defined by the number of baseline risk factors above clinically optimal levels (ranging from 0–5). Based on the National Institute for Health and Care Excellence guidance (evidence-based recommendations for health and care in the United Kingdom), we used the following risk factor thresholds to define suboptimal status among people with T2D: current smoker, total cholesterol >4 mmol/L, triglycerides >1.7 mmol/L, HbA1c (glycohemoglobin) ≥53 mmol/mol (7.0%), and systolic blood pressure >140 mm Hg or >130 mm Hg in the presence of renal impairment, retinopathy or cerebrovascular disease.^17^

### Statistical Analysis

Age-standardized incidence rates, using the European Standard Population (unweighted average of individual populations from all 27 European Union countries and 3 European Free Trade Association States),^18^ expressed per 100 person-years, were calculated for cardiovascular outcomes in controls and in people with T2D stratified by the number of risk factors above target levels and by presence/absence of cardio-renal disease (defined as prior myocardial infarction, stroke, CHD, or eGFR <45 mL/min/1.73m^2^).

In CPRD, we used Cox regression to examine risks for primary and secondary outcomes in relation to the number of risk factors above threshold values among people with T2D compared with matched controls. In CPRD and SCI-Diabetes T2D cohorts, Cox regression examined risks for CVD outcome associated with the number of risk factors above threshold values compared with people with optimal risk factor control (ie, no risk factors above threshold values). Cox models were adjusted for age, sex, deprivation, ethnicity, diabetes duration, and history of CVD. To account for matched cohort design, the Cox regression was stratified by matched sets. History of CVD was not included in the analyses stratified by cardio-renal disease. Risk estimates from the 2 cohorts were pooled by DerSimonian and Laird random-effects meta-analysis. A *P* value <0.05 was considered statistically significant. The Bonferroni adjustment was applied to the analysis of secondary end points, computing confidence intervals (CIs) and *P* values that account for multiple comparisons. As a sensitivity analysis, models were stratified by gender to assess for effect modification in the relationship between risk factor control and CVD outcomes.

Missing baseline data were imputed with the multivariate imputation by chained equations algorithm. Five complete data sets were imputed; variables used in imputation are provided in Methods in the Data Supplement.

All relevant code lists for variables and outcomes are publicly available at The ClinicalCodes repository^19^ and can be accessed at https://clinicalcodes.rss.mhs.ac.uk. Analyses were performed using Stata 15.1 (StataCorp LP, College Station, TX).

## Results

### Study Populations

Baseline characteristics of the study cohorts are shown in the Table, with additional detail provided in Table I in the Data Supplement (CPRD) and Table II in the Data Supplement (SCI-Diabetes). The CPRD cohort comprised of 101 749 people with T2D and 378 938 controls without diabetes with median (interquartile range) follow-up of 2.9 (1.3–5.3) years and 3.0 (1.3–5.4) years, respectively. A total of 73 096 (72%) people with T2D had complete data on all 5 risk factors and 68 815 (94%) individuals had at least 1 risk factor at a suboptimal level. The SCI-Diabetes cohort comprised of 330 892 individuals with T2D with a median (interquartile range) follow-up of 6.4 (3.1–9.2) years. A total of 201 653 (61%) people had complete data on all 5 risk factors and 189 404 (94%) individuals had at least 1 risk factor at a suboptimal level.

**Table 1.**
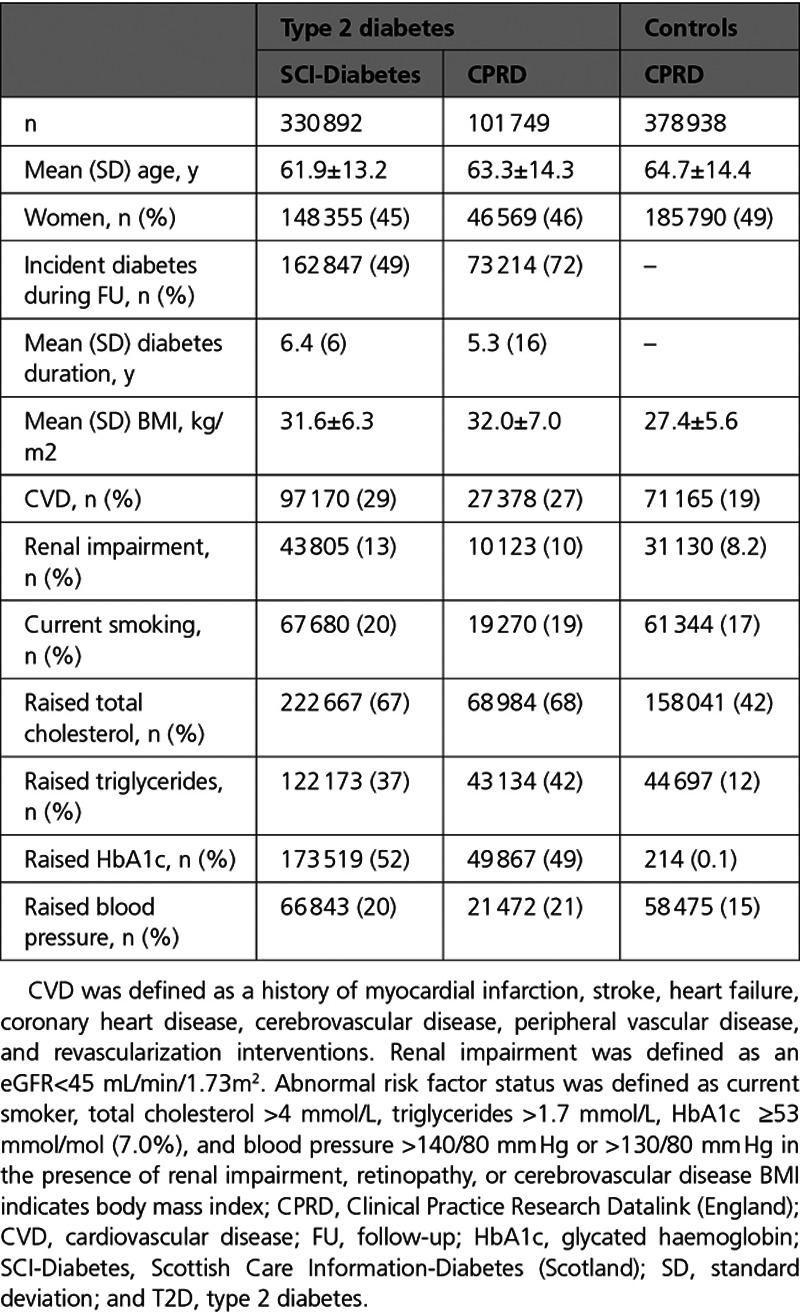
Baseline Clinical Characteristics of People With T2D From Scotland and of People With T2D and Controls From England

Clinical characteristics of the 2 cohorts were broadly similar. Compared with T2D patients in SCI-Diabetes, T2D patients in CPRD were slightly older, less likely to be current smokers, and less likely to have CVD and renal impairment, but had higher body mass index, total cholesterol, and HbA1c levels. Prescribing of antidiabetic, lipid-lowering, and antiplatelet medication were all lower in CPRD. Forty percent of participants were receiving glucose lowering medication reflecting the high proportion with newly diagnosed T2D (72%, CPRD; 49%, SCI-Diabetes). In both study cohorts, younger age was associated with a higher number of suboptimal risk factors present (Table I in the Data Supplement [CPRD] and Table II in the Data Supplement [SCI-Diabetes]).

Prevalent cardio-renal disease was identified in 28 933 (28%) people with type 2 diabetes in CPRD and 115 342 (35%) in SCI-Diabetes. Across CPRD and SCI-Diabetes, people with T2D and without prevalent cardio-renal disease (defined as at low risk of CVD) were on average 11 years younger than people with prevalent cardio-renal disease (mean age 58.9±13.2 versus 70.5±11.4 years) and were more likely to have a greater number of risk factors at suboptimal levels and fewer prescriptions for lipid-lowering medications, antiplatelet agents, and antihypertensive agents (Table III in the Data Supplement [CPRD] and Table IV in the Data Supplement [SCI-Diabetes]).

### Incidence Rates for Cardiovascular Events and Mortality

During the study period, cardiovascular events occurred in 27 900 (27%) patients with T2D in CPRD, 72 520 (19%) controls in CPRD, and in 101 362 (31%) patients with T2D in SCI-Diabetes. Cardiovascular death occurred in 3144 (3.1%) patients with T2D in CPRD, 10 131 (2.7%) controls in CPRD, and in 26 974 (8.1%) patients with T2D in SCI-Diabetes.

Age-standardized incidence rates for the cohorts are shown in Table V in the Data Supplement. In people with T2D with complete data on all 5 risk factors, the incidence of CVD events was generally higher in SCI-Diabetes compared with CPRD across all risk factor control levels, with the exception of heart failure hospitalization. A U-shaped relationship between degree of risk factor control and CVD incidence was observed in both cohorts when previous CVD history was not taken into account. Cardiovascular mortality was consistently higher in SCI-Diabetes.

Incidence rates were considerably higher in those with prevalent cardio-renal disease compared with those without cardio-renal disease (Table VI in the Data Supplement).

### Risk for Cardiovascular Events and Mortality

**Figure 2.**
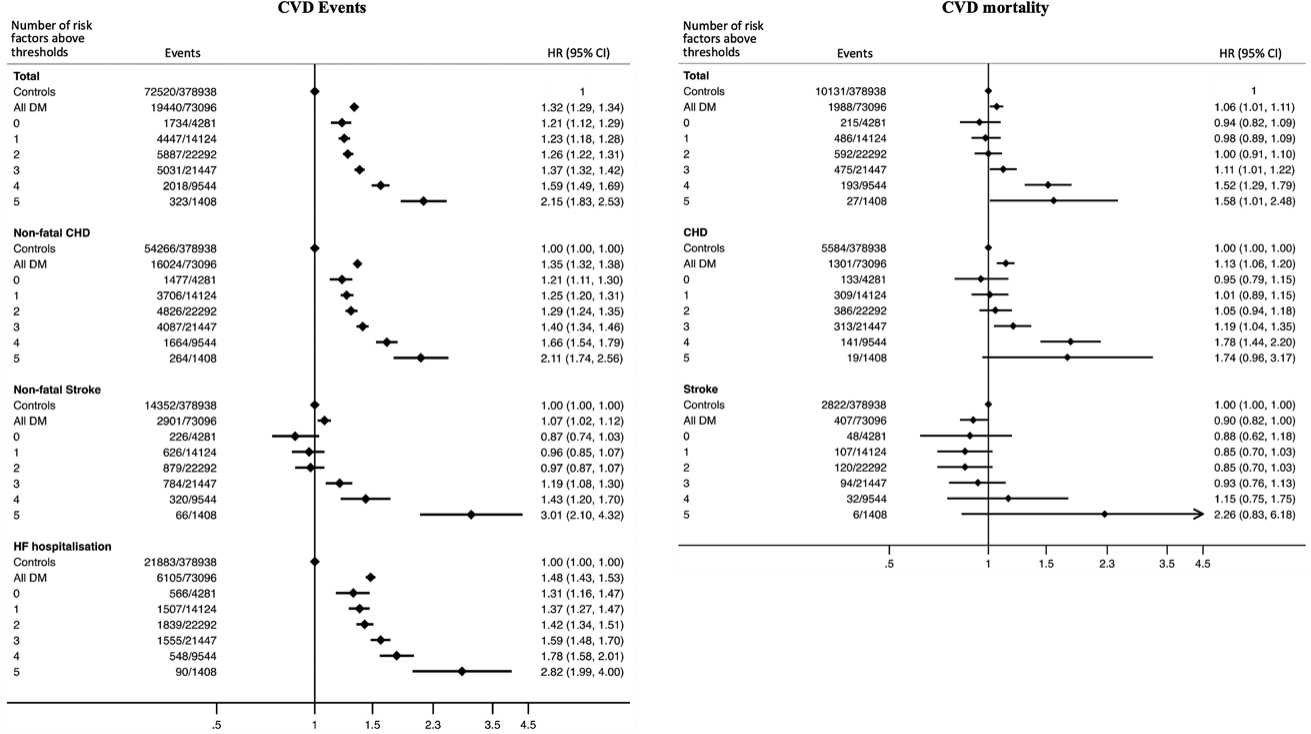
**Multivariable-adjusted relative hazards for cardiovascular disease (CVD) events (fatal or nonfatal CVD event or heart failure hospitalization) and mortality according to number of risk factors above thresholds in people with type 2 diabetes (CPRD [Clinical Practice Research Datalink]) compared to matched controls without diabetes (CPRD).** Adjusted for age, sex, deprivation, ethnicity, diabetes duration and history of CVD. Hazard ratios are pooled from all 5 data sets. Number of events and population represent the mean in the 5 data sets. CHD indicates coronary heart disease; CI, confidence interval; DM, diabetes; HF, heart failure; and HR, hazard ratio.

Figure 2 shows the adjusted hazard ratios (HRs) for cardiovascular outcomes associated with the number of risk factors above threshold values in CPRD T2D patients compared with controls without diabetes. Overall, people with T2D had an approximately 30% higher risk of cardiovascular events compared with controls after adjusting for age, sex, deprivation, ethnicity, and prevalent CVD.

Across all cardiovascular events, increasing numbers of risk factors above thresholds were associated with higher adjusted risks relative to controls. For people with T2D who had optimal risk factor control, the adjusted HR for CVD events was 1.21 (95% CI, 1.12–1.29) compared with controls. The corresponding adjusted HR was 2.15 (95% CI, 1.83–2.53) for those with all 5 risk factors above target levels. Cardiovascular mortality risk among people with T2D and 0 to 2 risk factors above targets was not significantly higher than controls. As the number of risk factors above target levels increased (3–5 risk factors), the risk for cardiovascular death increased and was significantly higher than controls.

**Figure 3.**
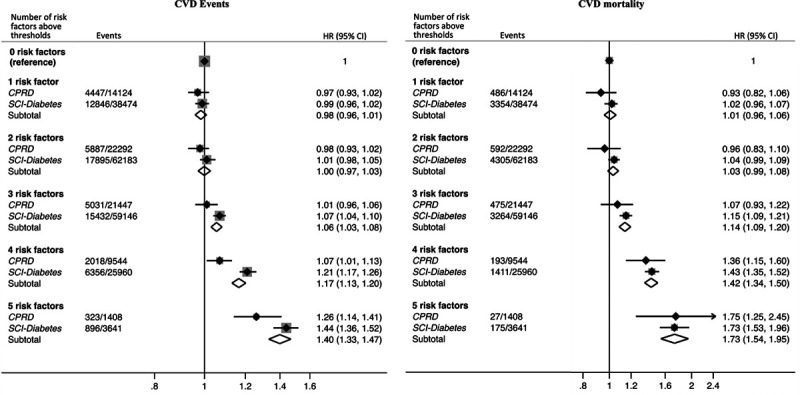
**Meta-analysis of multivariable-adjusted relative hazards for cardiovascular disease (CVD) events (fatal or nonfatal CVD event or heart failure hospitalization) and mortality according to number of risk factors above thresholds in people with type 2 diabetes from CPRD (Clinical Practice Research Datalink) and SCI-Diabetes (Scottish Care Information-Diabetes) compared to optimally controlled type 2 diabetes.** Adjusted for age, sex, deprivation, ethnicity, diabetes duration, and history of CVD. Hazard ratios are pooled from all 5 data sets. Number of events and population represent the mean in the 5 data sets. CI indicates confidence interval; and HR, hazard ratio.

Overall, compared to optimally controlled people with T2D in CPRD and SCI-Diabetes, those with T2D and 5 risk factors above target had pooled adjusted HRs of 1.40 (95% CI, 1.33–1.47) for total CVD events, 1.25 (95% CI, 1.18–1.32) for nonfatal CHD, 2.30 (95% CI, 2.02–2.63) for nonfatal stroke, 1.46 (95% CI, 1.31–1.63) for heart failure hospitalization, 1.73 (95% CI, 1.54–1.95) for total CVD morality, 1.84 (95% CI, 1.60–2.11) for CHD mortality, and 1.80 (95% CI, 1.33–2.43) for stroke mortality (Figure 3 [total CVD] and Figure II in the Data Supplement [individual CVD components]).

**Figure 4.**
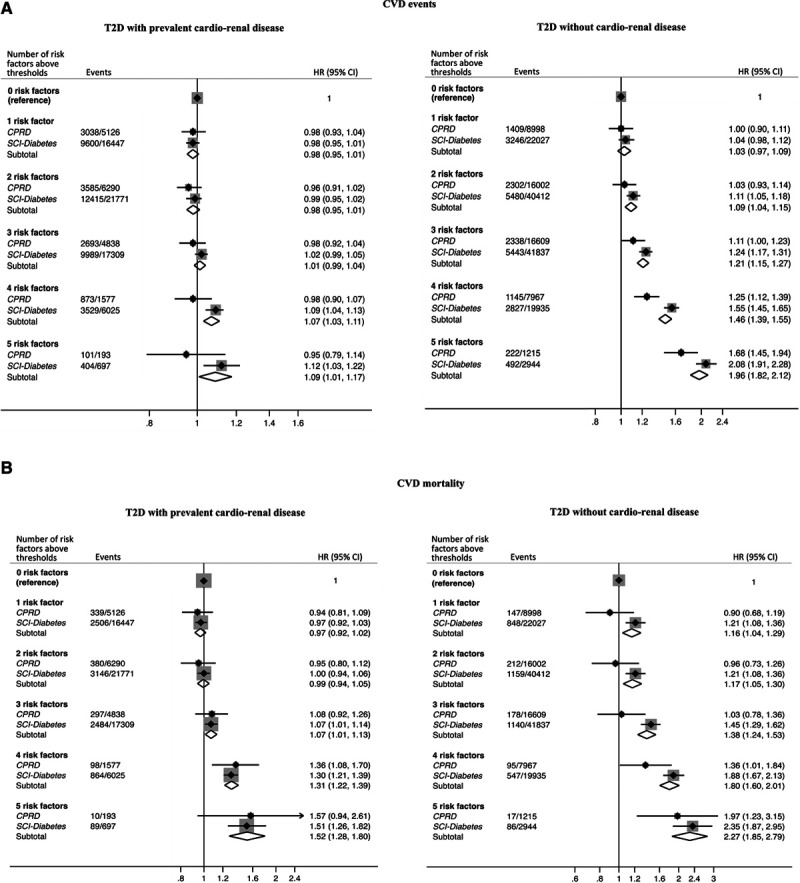
**Meta-analysis of multivariable-adjusted relative hazards for cardiovascular disease (CVD) events.** **A**, fatal or nonfatal CVD event or heart failure hospitalisation and (**B**), CVD mortality, according to number of risk factors above thresholds in people with type 2 diabetes from CPRD (Clinical Practice Research Datalink) and SCI-Diabetes (Scottish Care Information-Diabetes) compared with optimally controlled type 2 diabetes, stratified by the presence of cardio-renal disease. Cardio-renal disease defined as: prior history of acute myocardial infarction, stroke, coronary heart disease and/or renal impairment. Adjusted for age, sex, deprivation, ethnicity, and diabetes duration. Hazard ratios are pooled from all 5 data sets. Number of events and population represent the mean in the 5 data sets. CI indicates confidence interval; HR, hazard ratio; and T2D, type 2 diabetes.

In people with T2D and cardio-renal disease, the pooled association between the number of risk factors above target and risk of CVD events was weak (Figure 4A). There was no significant risk increase when 1 to 3 risk factors were above thresholds; however, when 4 and 5 risk factors were elevated, the respective risks were 7% (HR, 1.07; 95% CI, 1.03–1.11) and 9% (HR, 1.09; 95% CI, 1.01–1.17) higher than optimally controlled people. In contrast, in people with T2D and no cardio-renal disease, CVD risk increased stepwise for each additional risk factor above target, with a near 2-fold (HR, 1.96; 95% CI, 1.82–2.12) higher risk for those with 5 elevated risk factors (Figure 4A). A similar pattern was observed for CVD mortality with stronger associations with risk factor control in those without cardio-renal disease (Figure 4B). In people with T2D and cardio-renal disease, CVD mortality risk was modestly higher than in optimally controlled patients when 4 (HR, 1.31; 95% CI, 1.22–1.39) or 5 (HR, 1.52; 95% CI, 1.28–1.80) risk factors were above targets. However, in contrast, those without cardio-renal disease with 1 risk factor above target had a 16% (HR, 1.16; 95% CI, 1.04–1.29) higher CVD mortality risk compared with optimally controlled people. Each additional risk factor above target was associated with increasingly higher risks, with more than twice the risk observed in those with 5 risk factors above target (HR, 2.27; 95% CI, 1.85–2.79). A similar pattern was observed for the risks for individual nonfatal and fatal events (CHD, stroke, and heart failure hospitalization) associated with different levels of risk factor control in patients with and without cardio-renal disease (Figure IIIA in the Data Supplement [CVD events] and Figure IIIB in the Data Supplement [CVD mortality]).

In the sensitivity analysis, stratifying by gender had no material effect on the relationship between the degree of risk factor control and risk of CVD outcomes (results not shown).

## Discussion

### Key Findings

Our key findings with important clinical implications are: 1) compared with people without diabetes, those with T2D and optimally managed risk factors have a 21% higher risk for all CVD events and nonfatal CHD and a 31% higher risk for heart failure hospitalization; 2) only 6% of people with T2D had optimal risk factor control; and 3) the association between the number of elevated risk factors and CVD events/mortality was much stronger in people with T2D without cardio-renal disease when compared with T2D and established cardio-renal disease.

### Prior Studies

The landmark Steno-2 trial showed that intensive CVD risk factor intervention could halve risk for both CVD events and mortality in subjects with T2D and microalbuminuria.^3–6,20^ In an extended follow-up of the cohort, life expectancy was 8 years longer with intensive risk factor control.^5^ These impressive results show how simultaneous intervention on multiple risk factors might have major benefits in people with T2D. However, the optimal strategy for multifactorial intervention in T2D has not been established based on other multifactorial intervention trials,^21–24^ and meta-analyses,^25^ that have provided inconsistent results. It is useful to highlight that these trials were performed before SGLT2 inhibitors (SGLT2i) and GLP-1 receptor agonists (GLP-1RA) were in common clinical use. Since these agents improve CVD risk factors,^26–28^ and reduce CVD risk, intensive intervention using these agents may deliver even more impressive CVD benefits than the Steno-2 trial might suggest.^29,30^

Our observational data suggest that in people with T2D there is a clinically significant residual CVD risk even when all causal risk factors are optimally managed, at least to levels mandated in the United Kingdom by the National Institute for Health and Care Excellence ^17^; 21% to 31% higher risk for CVD events and heart failure hospitalization compared with people without diabetes. These findings are in contrast to the results from a large T2D Swedish study in which optimal risk factor control was not linked to higher risks when compared with the general population.^8^ Potential explanations for these discrepant results include: 1) differences in the risk factors studied, specifically regarding albuminuria and cholesterol; 2) differences in the health of participants without diabetes; and 3) differences in diabetes management including lifestyle interventions and type of diabetes medications.

As far as we are aware, no diabetes study has previously shown that the associations between risk factor levels and CVD outcomes were stronger in low-risk people without cardio-renal disease compared with high-risk individuals with cardio-renal disease. These findings have important clinical implications. Individuals with T2D and absence of cardio-renal disease were younger than T2D individuals with cardio-renal disease (mean age: 59 versus 71 years) and had higher CVD risk factors levels. The risk difference between the least and best-controlled low-risk individuals was ≈2-fold higher for both CVD events and for CVD mortality (Figure 4), suggesting the importance of optimal medical therapy in these people. This follows a similar pattern observed in data presented by Rawshani et al in which risks for death and cardiovascular outcomes increased dramatically in younger than in older people.^8^ These more pronounced risks in younger individuals without cardio-renal disease may be due to the age at which T2D was diagnosed. Younger-onset T2D poses greater excess CVD morbidity and mortality risk than later-onset T2D, highlighting the need for, and the potential gains from, more aggressive intervention.^31^

We can only speculate on the reasons why associations between risk factor levels and CVD outcomes were stronger in people without cardio-renal disease. Although differences in drug regimens and drug interactions may play a role, it seems plausible that different metabolic pathways could underlie the development and progression of intimal plaque atheroma seen in people without cardio-renal disease compared with the medial arterial calcification commonly seen in those with cardio-renal disease. Our data, and the disappointing results of clinical trials of CVD risk factor intervention in dialysis populations, support the idea that risk factor intervention needs to be initiated early, before CVD or stage 3b chronic kidney disease develops, in order to maximize the benefits of risk reduction by these means.^32^

We showed that in individuals with cardio-renal disease, risk factor levels appeared to contribute little to the relative risk for the combined fatal and nonfatal CVD event end point (9% relative risk between optimally-controlled and poorly controlled participants) but were strongly related to risk for fatal CVD (52% relative risk). Therefore, benefits from risk factor intervention in this group may be dominated by a reduction in risk for fatal CVD rather than nonfatal CVD.

### Clinical Implications

While the benefits of optimal risk factor control have been demonstrated (risk differences between the best- and least-controlled individuals with T2D was 40% for CVD events and 73% for CVD mortality) we observed that only 6% of people with T2D had optimal risk factor control. The issue of inadequate risk factor management in T2D is an international problem.^33–35^

Our data supports early and more intensive intervention in people with T2D who are perceived to be at lower risk (without established cardio-renal disease), whom on average, have higher body mass index, total cholesterol, HbA1c, and blood pressure levels than individuals with established cardio-renal disease. Such intervention could yield substantial long-term reductions in CVD events and mortality at the population level. In keeping with the results of the National Diabetes Audit,^35^ our data showed that a smaller proportion of patients without cardio-renal disease were receiving antihypertensive therapy and statins compared with people with cardio-renal disease (48% versus 80% and 37% versus 70%, respectively). We support calls for greater use of early guideline-driven care, wider use of newer agents including SGLT2i and GLP-1RA that lower cardio-renal risks beyond effects on HbA1c, pharmacist-led clinics,^36^ IT systems supporting self-management,^37^ and clinical decision support for clinical staff.^38^

While we emphasise the potential benefits of risk factor reduction in individuals without cardio-renal disease (considered to be low-risk), we would not want to minimize the potential importance of risk factor control in patients with cardio-renal disease (considered to be high-risk) for several reasons: 1) our data do not inform us about the potential benefits of treatment already given to individuals considered to be at high-risk (eg, ≈3 quarters were receiving antihypertensive and lipid-lowering therapy); 2) although our data predict modest further reductions in the relative risk for total CVD in people with cardio-renal disease through further risk factor intervention, the number needed to treat to prevent an event may be small due to high absolute risk for events; and 3) risk factors studied appeared to contribute significantly to the relative risk for fatal CVD events (52% relative risk between optimally controlled and poorly controlled participants).

### Strengths and Limitations

Our study strengths include: 1) combining linked data from 2 large T2D cohorts from England and Scotland; 2) having a control group without diabetes; 3) stratifying outcomes by baseline CVD risk, defined by prevalent cardio-renal disease; and 4) presenting results which are general to the UK population. We acknowledge these limitations: omission of albuminuria as a risk factor due to lack of data, and in CPRD, prevalent T2D cases were required to have a diabetes diagnostic code documented by the General Practice within the study window period (2006–2013). Since such coding may occur sometimes after hospitalization, these cases may have higher CVD risk than prevalent cases not satisfying this selection criterion. However, this limitation is unlikely to have substantially affected our results as the proportion of prevalent T2D in CPRD was 28%, and as shown, relationships between risk factor levels and CVD risk were similar between our CPRD and SCI-Diabetes cohorts, with the latter capturing the entire population in Scotland.

## Conclusions

Compared with people without diabetes, those with T2D have higher risks for CVD events, CVD mortality, and heart failure hospitalization even when all causal risk factors are optimally controlled to levels mandated in current clinical guidelines. We found that the association between risk factor levels and CVD outcomes was much stronger in people with T2D without cardio-renal disease when compared with those with cardio-renal disease at cohort entry. Since overall risk factor management was poor, we encourage greater use of guideline-driven care, newer agents including SGLT2i and GLP-1RA, pharmacist-led clinics, IT systems supporting self-management, and clinical decision to support for clinical staff.

## Acknowledgments

We acknowledge Jeremy Walker’s contribution to providing additional data for the SCI-Diabetes cohort. We acknowledge with gratitude the contributions of people with diabetes, NHS staff, and organizations (the Scottish Care Information-Diabetes Steering Group, the Scottish Diabetes Group, the Scottish Diabetes Survey Group, and the diabetes managed clinical networks) involved in providing data, setting up, maintaining and overseeing collation of data for people with diabetes in Scotland. The Scottish Diabetes Research Network is supported by National Health Service Research Scotland, a partnership involving Scottish NHS Boards and the Chief Scientist Office of the Scottish Government.

## Sources of Funding

This study was funded by Diabetes UK (BDA: 14/0004971). We acknowledge financial support from Medical Research Council (Health eResearch Center Grant MR/K006665/1 and recent Methodology awards; MR/T025085/1 and Wellcome C-GULL birth cohort grants 217067/Z/19/Z). The views expressed are those of the authors and not necessarily those of Diabetes UK. The funders of the study had no role in study design or in data collection, analysis, or interpretation.

## Disclosures

D.M.A. reports research funding from Abbvie, Almirall, Celgene, Eli Lilly, Novartis, UCB, and the Leo Foundation outside the submitted work. M.K.R. reports receiving research funding from Novo Nordisk, consultancy fees from Novo Nordisk and Roche Diabetes Care, and modest owning of shares in GlaxoSmithKline. The other authors have no conflicts of interest to disclose.

## Supplemental Materials

Expanded Methods

Data Supplement Figures I–III

Data Supplement Tables I–VI

Reference 39

## Supplementary Material


