# Supplementary material for: Risk Factor Control and Cardiovascular Event Risk in People With Type 2 Diabetes in Primary and Secondary Prevention Settings
Source: Circulation. 2020 Nov 17;142(20):1925–36. doi: 10.1161/CIRCULATIONAHA.120.046783 (PMC7664968; doi:10.1161/CIRCULATIONAHA.120.046783)
Supplement: Supplementary file 1 [file cir-142-1925-s001.pdf]

SUPPLEMENTAL MATERIAL

Table of Contents

Page

Supplemental Methods

Imputation 3

Supplemental Tables

Supplemental Table I. Baseline characteristics of people with type 2 diabetes by risk factor status and matched controls without diabetes in CPRD 4-5

Supplemental Table II. Baseline characteristics of people with type 2 diabetes in SCI-Diabetes by risk factor status 6-7

Supplemental Table III. Baseline characteristics of people with type 2 diabetes in CPRD, stratified by prevalent cardio-renal disease 8-9

Supplemental Table IV. Baseline characteristics of people with type 2 diabetes in SCI-Diabetes, stratified by prevalent cardio-renal disease 10-11

Supplemental Table V. Age-standardised rates for cardiovascular events (fatal or non-fatal CVD event or heart failure hospitalisation) and mortality in people with type 2 diabetes and controls 12

Supplemental Table VI. Age-standardised rates for cardiovascular events (fatal or non-fatal CVD event or heart failure hospitalisation) and mortality in people with type 2 diabetes and controls, stratified by the presence of cardio-renal disease 13

Supplemental Figures

Supplemental Figure I. Structure of hospital in-patient data in England and Scotland 14

Supplemental Figure II. Meta-analysis of multivariable-adjusted relative hazards for CHD, stroke and HF hospitalisation according to number of risk factors above thresholds in people with type 2 diabetes from CPRD and SCI-Diabetes compared to people with optimal risk factor control 15

Supplemental Figure III. Meta-analysis of multivariable-adjusted relative hazards for CHD, stroke and HF hospitalisation according to number of risk factors above thresholds in people with type 2 diabetes from CPRD and SCI-Diabetes compared to people with optimal risk factor control, stratified by the presence of cardio-renal disease 16-17

Supplemental Reference

Reference 39 18

Supplemental Methods

Imputation

We used multivariate imputation by chained equations (MICE) to impute 5 complete data sets with 100 iterations for the burn-in period for each data set. In CPRD, imputation models were constructed separately for people with type 2 diabetes and controls without diabetes.

Variables with missing data included; BMI, systolic blood pressure, diastolic blood pressure, HbA1c, triglycerides, total cholesterol, renal impairment and smoking status.

Variables included in the imputation model were; sex, age, deprivation, ethnicity, diabetes duration, calendar year, history of microvascular complications, history of myocardial infarction, history of stroke, history of coronary heart disease, history of cerebrovascular disease, history of revascularisations, use of antidiabetic medications, use of antihypertensive agents, use of lipid-lowering medications, use of antiplatelet agents, outcome for total cardiovascular events (fatal/non-fatal coronary heart disease, stroke, heart failure hospitalisation), outcome for non-fatal coronary heart disease, outcome for non-fatal stroke, outcome for heart failure hospitalisation, outcome for total cardiovascular mortality, outcome for coronary heart disease mortality, outcome for stroke mortality.

Supplemental Tables

Supplemental Table I. Baseline characteristics of people with type 2 diabetes by risk factor status and matched controls without diabetes in CPRD

	**Controls**	**Type 2 Diabetes patients**						
		**All T2D**	***No. of risk factors above threshold (with complete data on all factors: N=73,096)***					
			**0**	**1**	**2**	**3**	**4**	**5**
**N (%)**	378,938	101,749	4,281 (5.9)	14,124 (19)	22,292 (31)	21,447 (29)	9,544 (13)	1,408 (1.9)
**Men, n (%)**	193,148 (51)	55,180 (54)	2,741 (64)	7,854 (56)	11,558 (52)	11,736 (53)	5,526 (58)	927 (66)
**Age, years ***	64.7 ± 14.4	63.3 ± 14.3	70.3 ± 12.3	67.3 ± 13.0	64.0 ± 13.2	60.5 ± 13.3	56.9 ± 12.4	54.0 ± 10.8
**Duration of diabetes, years †**	-	5.3 (0.3-15.8)	10.7 (1.4-17.9)	9.0 (0.9-17.0)	5.7 (0.4-16.0)	2.8 (0.1-13.0)	1.0 (0.1-9.5)	0.4 (0.1-7.5)
**Newly diagnosed T2D** (0-3 months)	-	88,508 (87)	3,327 (78)	11,594 (82)	19,239 (86)	19,196 (90)	8,801 (92)	1,326 (94)
**Ethnicity**								
White	346,365 (91)	88,074 (87)	3,769 (88)	12,146 (86)	19,082 (86)	18,541 (87)	8,341 (87)	1,243 (88)
South Asian	6,984 (1.8)	5,569 (5.5)	219 (5.1)	797 (5.6)	1,344 (6.0)	1,256 (5.9)	481 (5.0)	57 (4.1)
Black	5,040 (1.3)	2,538 (2.5)	100 (2.3)	461 (3.3)	618 (2.8)	451 (2.1)	159 (1.7)	20 (1.4)
Other	3,679 (1.0)	1,365 (1.3)	53 (1.2)	180 (1.3)	323 (1.5)	279 (1.3)	122 (1.3)	22 (1.6)
Unknown	16,870 (4.5)	4,203 (4.1)	140 (3.3)	540 (3.8)	925 (4.2)	920 (4.3)	441 (4.6)	66 (4.7)
**Deprivation (IMD 2010)**								
IMD 1 (most deprived)	59,033 (16)	19,107 (19)	740 (17)	2,430 (17)	3,985 (18)	4,265 (20)	2,169 (23)	363 (26)
IMD 2	72,764 (19)	21,439 (21)	840 (20)	2,877 (20)	4,690 (21)	4,524 (21)	2,096 (22)	347 (25)
IMD 3	77,248 (20)	20,647 (20)	834 (20)	2,743 (19)	4,444 (20)	4,365 (20)	1,860 (20)	292 (21)
IMD 4	88,852 (24)	22,211 (22)	998 (23)	3,211 (23)	4,918 (22)	4,464 (21)	1,847 (19)	248 (18)
IMD 5 (least deprived)	80,768 (21)	18,277 (18)	864 (20)	2,855 (20)	4,2447 (19)	3,810 (18)	1,565 (16)	157 (11)
Unknown	273 (0.1)	68 (0.1)	5 (0.1)	8 (0.1)	11 (0.1)	19 (0.1)	7 (0.1)	1 (0.1)
**Duration of follow-up, years ***	3.5 ± 2.5	3.4 ± 2.5	3.7 ± 2.6	3.7 ± 2.5	3.7 ± 2.5	3.8 ± 2.5	3.9 ± 2.5	3.8 ± 2.5
**Duration of follow-up, years †**	3.0 (1.3-5.4)	2.9 (1.3-5.3)	3.2 (1.4-5.5)	3.2 (1.5-5.6)	3.3 (1.6-5.7)	3.4 (1.7-5.7)	3.6 (1.8-5.9)	3.5 (1.7-5.8)
**Comorbidities**								
**History of CVD**	71,165 (19)	27,378 (27)	2,104 (49)	4,887 (35)	5,821 (26)	4,533 (21)	1,535 (16)	202 (14)
Myocardial infarction	17,852 (4.7)	8,000 (7.9)	722 (17)	1,545 (11)	1,749 (7.9)	1,239 (5.8)	374 (3.9)	41 (2.9)
Stroke	14,938 (3.9)	5,410 (5.3)	403 (9.4)	879 (6.2)	1,051 (4.7)	779 (3.6)	311 (3.3)	48 (3.4)
Coronary heart disease	47,408 (13)	19,681 (19)	1,621 (38)	3,651 (26)	4,269 (19)	3,262 (15)	988 (10)	119 (8.5)
Cerebrovascular disease	25,570 (6.8)	8,648 (8.5)	631 (15)	1,462 (10)	1,708 (7.7)	1,273 (5.9)	479 (5.0)	68 (4.8)
Cardiac revascularisation	19,015 (5.0)	8,734 (8.6)	794 (19)	1,721 (12)	1,897 (8.5)	1,416 (6.6)	449 (4.7)	41 (2.9)
**Renal impairment** (eGFR <45ml/min/1.73m$_2$)	31,130 (8.2)	10,123 (10)	693 (16)	1,765 (13)	2,264 (10)	1,775 (8.3)	531 (5.6)	43 (3.1)
**Risk Factors**								
**Current Smoker**	61,344 (68)	19,270 (19)	0	767 (5.4)	2,693 (12)	5,129 (24)	5,303 (56)	1,408 (100)
unknown (%)	*32*	*11*	-	-	-	-	-	-
**BMI (kg/m$_2$)***	27.4 ± 5.6	32.0 ± 7.0	29.8 ± 6.4	30.5 ± 6.6	31.4 ± 6.8	32.4 ± 6.8	33.3 ± 7.1	33.8 ± 7.1

missing (%)	*67*	*38*	*4.2*	*3.6*	*3.4*	*3.4*	*3.5*	*3.5*
**Total cholesterol (mmol/L) ***	5.0 ± 1.1	5.0 ± 1.3	3.4 ± 0.4	4.3 ± 1.0	4.9 ± 1.1	5.4 ± 1.2	5.7 ± 1.2	5.9 ± 1.3
missing (%)	*63*	*27*	-	-	-	-	-	-
**Triglycerides (mmol/L) ***	1.5 ± 0.8	2.1 ± 1.5	1.1 ± 0.3	1.3 ± 0.5	1.9 ± 1.1	2.6 ± 1.6	3.2 ± 1.9	3.5 ± 2.1
missing (%)	*60*	*24*	-	-	-	-	-	-
**HbA1c (mmol/mol) ***	39 ± 5	63 ± 22	45 ± 5	51 ± 14	57 ± 19	68 ± 23	74 ± 23	77 ± 22
missing (%)	*96*	*51*	-	-	-	-	-	-
**Blood pressure (mmHg) ***								
sBP	135 ± 16	138 ± 17	131 ± 15	133 ± 15	135 ± 16	138 ± 18	145 ± 19	156 ± 14
dBP	78 ± 10	80 ± 11	73 ± 9	76 ± 9	78 ± 10	81 ± 11	87 ± 11	93 ± 9
missing (%)	*38*	*18*	-	-	-	-	-	-
**Drug Prescriptions**								
**Diabetes**								
Any, %	-	33,284 (33)	1,411 (33)	4,513 (32)	7,265 (33)	7,985 (37)	3,784 (40)	562 (40)
Metformin, %	-	27,107 (27)	1,065 (25)	3,595 (26)	5,959 (27)	6,831 (32)	3,378 (35)	510 (36)
Sulphonylurea, %	-	7,677 (7.6)	476 (11)	1,289 (9.1)	1,620 (7.3)	1,509 (7.0)	583 (6.1)	69 (4.9)
Insulin, %	-	4,416 (4.3)	190 (4.4)	738 (5.2)	1,032 (4.6)	782 (3.7)	246 (2.6)	21 (1.5)
Other, %	-	2,678 (2.6)	174 (4.1)	526 (3.7)	639 (2.9)	515 (2.4)	170 (1.8)	28 (2.0)
**Antihypertensive agent**								
Any, %	142,174 (38)	55,974 (55)	3,299 (77)	9,591 (68)	13,580 (61)	11,614 (54)	4,629 (49)	689 (49)
Alpha-blocker, %	10,853 (2.9)	4,714 (4.6)	435 (10)	1,018 (7.2)	1,080 (4.8)	837 (3.9)	273 (2.9)	37 (2.6)
Angiotensin-2 receptor blocker, %	24,172 (6.4)	10,532 (10)	702 (16)	1,983 (14)	2,632 (12)	2,118 (9.9)	736 (7.7)	92 (6.5)
ACE inhibitor, %	60,722 (16)	28,855 (28)	1,777 (42)	4,953 (35)	6,870 (31)	6,070 (28)	2,530 (27)	411 (29)
Beta-blocker, %	47,090 (12)	19,759 (19)	1,420 (33)	3,645 (26)	4,847 (22)	3,908 (18)	1,356 (14)	176 (13)
Calcium channel blocker, %	54,766 (15)	22,764 (22)	1,435 (34)	4,246 (30)	5,684 (26)	4,565 (21)	1,772 (19)	269 (19)
Diuretic: thiazide, potassium sparing or loop, %	63,906 (17)	26,438 (26)	1,529 (36)	4,556 (32)	6,422 (29)	5,379 (25)	1,946 (20)	236 (17)
**Lipid lowering therapy**								
Any, %	87,149 (23)	44,101 (43)	3,184 (74)	7,948 (56)	10,656 (48)	9,127 (43)	3,706 (39)	527 (37)
**Antiplatelets**								
Any, %	62,296 (16)	25,848 (25)	2,079 (49)	5,031 (36)	6,162 (28)	4,752 (22)	1,686 (18)	200 (14)

Data presented as n(%), mean±SD * or median (25_{th}-75_{th} percentile) †, where indicated

Supplemental Table II. Baseline characteristics of people with type 2 diabetes in SCI-Diabetes by risk factor status

	Type 2 Diabetes patients						
	All T2D	*No. of risk factors above threshold (with complete data on all factors: N=201,653)*					
		0	1	2	3	4	5
**N**	330,892	12,249(6.1)	38,474 (19)	62,183 (31)	59,146 (29)	25,960 (13)	3,641 (1.8)
**Men, n (%)**	182,537 (55)	7,643(62)	21,359 (56)	33,660 (54)	32,743 (55)	15,117 (58)	2,217 (61)
**Age, years ***	61.9 ± 13.2	67.9 ± 11.3	65.1 ± 12.2	61.8 ± 12.5	58.5 ± 12.5	55.1 ± 11.8	52.7 ± 10.6
**Duration of diabetes, years †**	6.4 (3-10)	5.7 (3-10)	6.0 (3-11)	6.0 (3-11)	5.8 (3-11)	5.7 (3-10)	5.5 (3-9)
**Ethnicity**							
White	239,605 (72)	8,806 (72)	27,772 (72)	45,393 (73)	43,457 (74)	19,423 (75)	2,770 (76)
South Asian	7,826 (2.3)	324 (2.6)	945 (2.5)	1,589 (2.6)	1,515 (2.6)	637 (2.5)	95 (2.6)
Black	10,736 (3.2)	328 (2.7)	1,330 (3.5)	2,174 (3.5)	2,017 (3.4)	741 (2.9)	71 (2.0)
Other	1,035 (0.3)	38 (0.3)	101 (0.3)	199 (0.3)	147 (0.2)	72 (0.3)	8 (0.2)
Unknown	71,690 (22)	2,753 (23)	8,326 (22)	12,828 (21)	12,010 (20)	5,087 (20)	697 (19)
**Deprivation (SIMD 2010)**							
SIMD 1 (most deprived)	80,108 (24)	2,895 (24)	9,026 (24)	15,149 (24)	15,656 (27)	7,961 (31)	1,269 (35)
SIMD 2	76,056 (23)	2,788 (23)	8,677(23)	13,948 (22)	13,710 (23)	6,226 (24)	888 (24)
SIMD 3	66,365 (21)	2,369 (19)	7,389 (19)	12,084 (19)	11,366 (19)	4,712 (18)	675 (19)
SIMD 4	59,440 (18)	2,138 (18)	6,807 (18)	11,200 (18)	9,916 (17)	3,926 (15)	487 (13)
SIMD 5 (least deprived)	47,869 (14)	2,032 (17)	6,468 (17)	9,639 (16)	8,318 (14)	3,034 (12)	315 (8.7)
Unknown	1,054 (0.3)	27 (0.2)	107 (0.3)	163 (0.3)	180 (0.3)	101 (0.4)	7 (0.2)
**Duration of follow-up, years ***	5.9 ± 9.2	5.6 ± 3.2	5.7 ± 3.1	5.7 ± 3.1	5.7 ± 3.1	5.6 ± 3.1	5.5 ± 3.0
**Duration of follow-up, years †**	6.4 (3.1-9.2)	5.7 (2.6-9.2)	6.0 (2.8-9.2)	6.0 (2.8-9.2)	5.9 (2.8-9.2)	5.7 (2.8-9.1)	5.5 (2.8-8.7)
**Comorbidities**							
**History of CVD**	97,170 (29)	5,849 (48)	14,007 (36)	18,187 (29)	14,585 (25)	5,227 (20.)	637 (18)
Myocardial infarction	46,151 (14)	3,043 (25)	6,978 (18)	8,871 (14)	7,120 (12)	2,428 (9.3)	277 (7.6)
Stroke	21,377 (6.4)	1,209 (9.8)	2,881 (7.5)	3,586 (5.7)	3,040 (5.1)	1,172 (4.5)	168 (4.6)
Coronary heart disease	80,966 (24)	5,011 (41)	11,905 (31)	15,415 (25)	12,214(21)	4,245 (16)	484 (13)
Cerebrovascular disease	25,356 (7.6)	1,401 (11)	3,386 (8.8)	4,291 (6.9)	3,574 (6.0)	1,384 (5.3)	201 (5.5)
Cardiac revascularisation	25,314 (7.6)	1,815 (15)	4,058 (11)	5,178 (8.3)	4,016 (6.8)	1,358 (5.2)	156 (4.3)
**Renal impairment** (eGFR <45ml/min/1.73m2)	43,805 (13)	2,448 (20)	6,249 (16)	8,248 (13)	6,376 (13)	1,918 (9.0)	167 (5.9)
**Risk Factors**							
**Current Smoker**	67,680 (20)	0	2,682 (7.0)	8,555 (14)	14,567 (25)	14,262 (55)	3,641 (100)
unknown (%)	19,310 (5.8)	-	-	-	-	-	-
**BMI (kg/m2) ***	31.6 ± 6.3	30.2 ± 5.9	30.8 ± 6.3	31.5 ± 6.3	32.2 ± 6.2	33.8 ± 6.3	33.1 ± 6.2
missing (%)	21	13	13	14	15	16	16
**Total cholesterol (mmol/L) ***	4.7 ± 1.2	3.4 ± 0.4	4.1 ± 0.9	4.7 ± 1.0	5.2 ± 1.1	5.6 ± 1.2	5.8 ± 1.2
missing (%)	5.1	-	-	-	-	-	-
**Triglycerides (mmol/L) ***	2.3 ± 1.5	1.1 ± 0.3	1.4 ± 0.6	2.0 ± 1.1	2.8 ± 1.6	3.3 ± 1.9	3.7 ± 2.1
missing (%)	37	-	-	-	-	-	-
**HbA1c (mmol/mol) ***	62± 20	46 ± 5	53 ± 14	50 ± 18	69 ± 22	76 ± 22	78 ± 19

missing (%)	5.4	-	-	-	-	-	-
**Blood pressure (mmHg) ***							
sBP	137 ± 17	131 ± 14	133 ± 15	135 ± 16	138 ± 17	145 ± 19	156 ± 14
dBP	78 ± 10	72 ± 8	74 ± 9	76 ± 9	79 ± 10	85 ± 11	92 ± 8
missing (%)	3.7	-	-	-	-	-	-
**Drug Prescriptions**							
**Diabetes**							
Any, %	132,058 (40)	4,997 (41)	15,328 (40)	23,757 (38)	22,221 (38)	9,193 (35)	1,112 (31)
Metformin, %	87,754 (27)	3,167 (26)	10,021 (26)	15,898 (26)	15,394 (26)	6,607 (26)	836 (23)
Sulphonylurea, %	53,466 (16)	2,187 (18)	6,309 (16)	9,486 (15)	8,489 (14)	3,323 (13)	372 (10)
Insulin, %	25,167 (7.6)	696 (5.7)	3,018 (7.8)	5,227 (8.4)	5,129 (8.7)	1,924 (7.4)	195 (5.4)
Other, %	17,949 (5.4)	643 (5.2)	2,026 (5.3)	3,181 (5.1)	3,000 (5.1)	1,291 (5.0)	140 (3.8)
**Antihypertensive agent**							
Any, %	203,336 (62)	9,646 (79)	27,374 (71)	40,519 (65)	34,487 (58)	13,384 (52)	1,793 (49)
Alpha-blocker, %	16,128 (4.9)	1,020 (8.3)	2,469 (6.4)	3,082 (5.0)	2,285 (3.9)	820 (3.2)	112 (3.1)
Angiotensin-2 receptor blocker, %	33,823 (10)	1,750 (14)	4,950 (13)	7,086 (11)	5,698 (9.6)	2,053 (7.9)	268 (7.4)
ACE inhibitor, %	109,729 (33)	5,356 (44)	16,430 (38)	21,414 (34)	18,569 (31)	7,344 (28)	1,005 (28)
Beta-blocker, %	88,256 (27)	4,728 (39)	12090 (31)	17,798 (29)	14,653 (25)	5,256 (20)	634 (17)
Calcium channel blocker, %	78,585 (24)	4204 (34)	11,182 (29)	15,752 (25)	12,825 (22)	4,899 (19)	680 (19)
Diuretic: thiazide, potassium sparing or loop, %	68,475 (21)	3,001 (25)	9,215 (24)	13,832 (22)	11,706 (20)	4,525 (17)	591 (16)
**Lipid lowering therapy**							
Any, %	168,299 (51)	9,321 (76)	23,714 (62)	33,148 (53)	27,440 (46)	10,482 (40)	1,401 (39)
**Antiplatelets**							
Any, %	121,636 (37)	6,891 (56)	17,582 (46)	23,599 (38)	18,681 (32)	6,799 (26)	827 (23)

Data presented as n(%), mean±SD * or median (25_{th}-75_{th} percentile) †, where indicated

Supplemental Table III. Baseline characteristics of people with type 2 diabetes in CPRD, stratified by prevalent cardio-renal disease

	Type 2 Diabetes patients (N=101,749)													
	Overall		*No. of risk factors above threshold (with complete data on all factors: N=73,096)*											
			0<br>N= 4,281		1<br>N=14,124		2<br>N=22,292		3<br>N=21,447		4<br>N=9,544		5<br>N=1,408	
	**With cardio-renal disease**	**Without cardio-renal disease**	**With cardio-renal disease**	**Without cardio-renal disease**	**With cardio-renal disease**	**Without cardio-renal disease**	**With cardio-renal disease**	**Without cardio-renal disease**	**With cardio-renal disease**	**Without cardio-renal disease**	**With cardio-renal disease**	**Without cardio-renal disease**	**With cardio-renal disease**	**Without cardio-renal disease**
**N**	28,933	72,816	2,163	2,118	5,126	8,998	6,290	16,002	4,838	16,609	1,577	7,967	193	1,215
**Men, n (%)**	16,360 (57)	38,820 (53)	1,472 (68)	1,275 (60)	3,166 (62)	4,688 (52)	3,481 (55)	8,077 (51)	2,628 (54)	9,108 (55)	870 (55)	4,656 (58)	118 (61)	809 (67)
**Age, years ***	71.7 ± 11.6	59.9 ± 13.9	74.2 ± 10.0	66.3 ± 13.2	73.2 ± 10.2	64.0 ± 13.2	71.2 ± 10.9	61.1 ± 13.0	69.0 ± 11.4	58.0 ± 12.7	65.5 ± 11.4	55.2 ± 11.9	60.1 ± 10.7	53.0 ± 10.5
**Duration of diabetes, years †**	11.5 (1.3-18.2)	2.9 (0.1-12.8)	12.7 (2.1-19.2)	7.0 (1.0-17.1)	12.7 (3.0-18.8)	4.8 (0.4-15.4)	11.0 (1.3-18.2)	3.4 (0.2-13.5)	10.3 (0.7-17.4)	1.3 (0.1-9.5)	6.2 (0.3-14.8)	0.4 (0.1-6.2)	5.2 (0.2-14.3)	0.2 (0.1-5.5)
**Ethnicity**														
White	26,470 (92)	61,604 (85)	1,983 (92)	1,786 (84)	4,650 (91)	7,496 (83)	5,750 (91)	13,332 (83)	4,446 (92)	14,095 (85)	1,459 (93)	6,882 (86)	176 (91)	1,067 (88)
South Asian	1,088 (3.8)	4,481 (6.2)	84 (3.9)	135 (6.4)	204 (4.0)	593 (6.6)	244 (3.9)	1,100 (6.9)	177 (3.7)	1,079 (6.5)	57 (3.6)	424 (5.3)	4 (2.1)	53 (4.4)
Black	411 (1.4)	2,127 (2.9)	33 (1.5)	67 (3.2)	95 (1.9)	366 (4.1)	82 (1.3)	536 (3.4)	66 (1.4)	385 (2.3)	14 (0.9)	145 (1.8)	3 (1.6)	17 (1.4)
Other	255 (0.9)	1,110 (1.5)	18 (0.8)	35 (1.7)	45 (0.9)	135 (1.5)	56 (0.9)	267 (1.7)	41 (0.9)	238 (1.4)	10 (0.6)	112 (1.4)	3 (1.6)	19 (1.6)
Unknown	709 (2.5)	3,494 (4.8)	45 (2.1)	95 (4.5)	132 (2.6)	408 (4.5)	158 (2.5)	767 (4.8)	108 (2.2)	812 (4.9)	37 (2.4)	404 (5.1)	7 (3.6)	59 (4.9)
**Deprivation (IMD 2010)**														
IMD 1 (most deprived)	5,365 (19)	13,742 (19)	370 (17)	370 (17)	901 (17)	1,529 (17)	1,147 (18)	2,838 (18)	984 (20)	3,281 (20)	384 (24)	1,785 (22)	62 (32)	301 (25)
IMD 2	6,106 (21)	15,333 (21)	428 (20)	412 (20)	1,057 (21)	1,820 (20)	1,341 (21)	3,349 (21)	1,040 (22)	3,484 (21)	341 (22)	1,755 (22)	51 (26)	296 (24)
IMD 3	5,839 (20)	14,808 (20)	415 (19)	419 (20)	976 (19)	1,767 (20)	1,258 (20)	3,186 (20)	974 (20)	3,391 (20)	287 (18)	1,573 (20)	37 (19)	255 (21)
IMD 4	6,482 (22)	15,729 (22)	525 (24)	473 (22)	1,185 (23)	2,026 (22)	1,389 (22)	3,529 (22)	1,000 (21)	3,464 (21)	313 (20)	1,534 (19)	35 (18)	213 (18)
IMD 5 (least deprived)	5,115 (18)	13,162 (18)	423 (20)	441 (21)	1,003 (20)	1,852 (21)	1,150 (18)	3,094 (19)	833 (17)	2,977 (18)	252 (16)	1,313 (17)	8 (4)	149 (12)
Unknown	26 (0.1)	42 (0.1)	2 (0.1)	3 (0.1)	4 (0.1)	4 (0.04)	5 (0.1)	6 (0.04)	7 (0.1)	12 (0.10)	0	7 (0.1)	0	1 (0.1)
**Duration of follow-up, years ***	3.2 ± 2.5	3.5 ± 2.5	3.5 ± 2.5	3.8 ± 2.6	3.5 ± 2.5	3.8 ± 2.5	3.6 ± 2.5	3.8 ± 2.5	3.6 ± 2.5	3.8 ± 2.5	3.8 ± 2.5	3.9 ± 2.5	3.7 ± 2.4	3.8 ± 2.5
**Duration of follow-up, years †**	2.6 (1.1-4.9)	3.0 (1.3-5.4)	3.0 (1.4-5.3)	3.3 (1.5-5.8)	3.0 (1.4-5.3)	3.4 (1.6-5.7)	3.1 (1.5-5.4)	3.4 (1.6-5.8)	3.2 (1.5-5.5)	3.5 (1.7-5.8)	3.4 (1.6-5.7)	3.6 (1.8-5.9)	3.3 (1.7-5.5)	3.6 (1.7-5.8)
**Risk Factors**														
**Current Smoker**	4,379 (15)	14,891 (21)	0	0	330 (6.4)	437 (4.9)	819 (13)	1,874 (12)	1,145 (24)	3,984 (24)	873 (55)	4,430 (56)	193 (100)	1,215 (100)
unknown (%)	*2,941 (10)*	*7,955 (10)*	-	-	-	-	-	-	-	-	-	-	-	-
**BMI ***	30.5 ± 6.4	32.1 ± 7.1	29.3 ± 6.0	30.4 ± 6.7	29.8 ± 6.1	30.9 ± 6.9	30.7 ± 6.2	31.7 ± 7.0	31.4 ± 6.1	32.6 ± 7.0	32.4 ± 6.6	33.4 ± 7.1	34.3 ± 7.6	33.8 ± 7.0
missing (%)	*14.0*	*12.4*	*5.2*	*3.3*	*4.6*	*3.0*	*4.5*	*2.9*	*5.1*	*3.0*	*5.1*	*3.1*	*5.2*	*3.2*
**Total cholesterol ***	4.4 ± 1.2	5.2 ± 1.3	3.4 ± 0.5	3.5 ± 0.4	4.0 ± 0.9	4.5 ± 1.1	4.5 ± 1.0	5.1 ± 1.1	5.0 ± 1.1	5.5 ± 1.2	5.3 ± 1.1	5.8 ± 1.2	5.5 ± 1.1	6.0 ± 1.3
missing (%)	*8.5*	*10.7*	-	-	-	-	-	-	-	-	-	-	-	-
**HbA1c (mmol/mol) ***	58 ± 19	62 ± 22	45 ± 5	45 ± 5	52 ± 15	51 ± 14	57 ± 18	57 ± 20	66 ± 22	68 ± 23	71 ± 21	75 ± 23	74 ± 23	78 ± 21
missing (%)	*9.0*	*10.8*	-	-	-	-	-	-	-	-	-	-	-	-
**Blood pressure ***														

sBP	135 ± 18	138 ± 17	131 ± 16	132 ± 14	133 ± 16	133 ± 15	135 ± 17	135 ± 16	138 ± 18	139 ± 18	145 ± 20	145 ± 19	154 ± 15	157 ± 14
dBP	75 ± 11	81 ± 11	71 ± 9	75 ± 8	73 ± 9	77 ± 9	75 ± 10	79 ± 9	78 ± 11	82 ± 10	84 ± 11	87 ± 11	91 ± 8	94 ± 9
missing (%)	*5.5*	*8.2*	-	-	-	-	-	-	-	-	-	-	-	-
**Drug Prescriptions**														
**Diabetes**														
Any oral hyoglycaemic agent, %	35	32	33	33	35	30	36	31	39	37	41	39	41	40
Metformin, %	25	28	23	27	25	26	26	27	29	33	33	36	37	36
Sulphonylurea, %	11	6.3	12	10	12	7.4	10	6.2	10	6.2	9.2	5.5	7.3	4.5
Insulin, %	7.5	3.1	4.6	4.3	7.3	4.1	7.8	3.4	7.7	2.5	6.4	1.8	4.7	1.0
Other, %	3.5	2.3	4.0	4.2	4.4	3.3	3.8	2.5	3.8	2.0	3.3	1.5	3.1	1.8
**Antihypertensive agent**														
Any, %	80	45	89	65	88	57	86	51	84	45	80	42	72	45
Alpha-blocker, %	7.3	3.6	11	9.1	9.4	6.0	7.4	3.9	6.5	3.1	6.5	2.1	4.7	2.3
Angiotensin-2 receptor blocker, %	16	8.1	19	14	19	11	18	9.5	17	7.9	15	6.2	13	5.5
ACE inhibitor, %	43	23	50	32	48	28	45	25	45	23	43	23	37	28
Beta-blocker, %	40	11	48	18	47	14	44	13	42	11	38	9.5	34	9.1
Calcium channel blocker, %	32	19	38	29	37	26	35	22	34	18	32	16	31	17
Diuretic: thiazide, potassium sparing or loop, %	40	20	42	30	43	26	42	24	42	20	38	17	30	15
**Lipid lowering therapy**														
Any, %	67	34	86	62	78	44	72	38	70	35	38	33	64	33
**Antiplatelets**														
Any, %	55	14	68	29	63	20	59	16	56	13	53	11	44	9.5

Data presented as n(%), mean±SD * or median (25_{th}-75_{th} percentile) †, where indicated

Supplemental Table IV. Baseline characteristics of people with type 2 diabetes in SCI-Diabetes, stratified by prevalent cardio-renal disease

	**Type 2 Diabetes patients (N=330,892)**													
	**Overall**		***No. of risk factors above threshold (with complete data on all factors: N=201,653)***											
			**0 N= 12,249**		**1 N=38,474**		**2 N=62,183**		**3 N=59,146**		**4 N=25,960**		**5 N=3,641**	
	**With cardio-renal disease**	**Without cardio-renal disease**	**With cardio-renal disease**	**Without cardio-renal disease**	**With cardio-renal disease**	**Without cardio-renal disease**	**With cardio-renal disease**	**Without cardio-renal disease**	**With cardio-renal disease**	**Without cardio-renal disease**	**With cardio-renal disease**	**Without cardio-renal disease**	**With cardio-renal disease**	**Without cardio-renal disease**
**N**	115,342	215,550	6,671	5,578	16,447	22,027	21,771	40,412	17,309	41,837	6,025	19,935	697	2,944
**Men, n (%)**	61,886 (54)	120,651 (56)	4,249(64)	3,394 (61)	9,440 (57)	11,919(54)	11,715 (54)	21,945(54)	8,973 (52)	23,770(57)	3,181 (53)	11,936 (60)	393 (56)	1,824 (62)
**Age, years ***	69.3 ± 11.1	57.9 ± 12.5	71.4 ± 9.8	63.8 ± 11.7	70.2 ± 10.2	61.1 ± 12.2	68.3 ± 10.6	58.2 ± 12.0	66.2 ± 11.0	55.3 ± 11.7	62.7 ± 11.2	52.8 ± 11.0	58.5 ± 10.1	51.3 ± 10.2
**Duration of diabetes, years †**	7 (4-12)	5 (3-9)	6 (3-11)	5 (3-9)	7 (4-13)	5 (3-9)	7 (4-13)	5 (3-9)	8 (4-13)	5 (3-9)	7 (4-12)	5 (2-9)	7 (3-11)	5 (2-8)
**Ethnicity**														
White	82,282 (71)	157,323 (73)	4,762 (71)	4,044 (73)	11,913 (72)	15,859 (72)	15,997 (74)	29,386 (73)	12,794 (74)	30,663 (73)	4,571 (76)	14,852 (75)	545 (78)	2,225 (76)
South Asian	2,531 (2.1)	5,295 (2.4)	176 (2.6)	148 (2.7)	366 (2.3)	579 (2.6)	524 (2.4)	1,065 (2.6)	440 (2.5)	1,075 (2.6)	151 (2.5)	486 (2.4)	15 (2.1)	80 (2.7)
Black	2,228 (1.9)	8,508 (3.9)	123 (1.8)	205 (3.7)	358 (2.2)	972 (4.4)	463 (2.1)	1,711 (4.2)	367 (2.1)	1,711 (4.2)	122 (2.0)	619 (3,1)	8 (1.2)	63 (2.1)
Other	111 (0.1)	924 (0.4)	7 (0.1)	31 (0.5)	15 (0.1)	86 (0.4)	25 (0.1)	174 (0.4)	22 (0.1)	174 (0.4)	3 (0.1)	69 (0.3)	2 (0.3)	6 (0.2)
Unknown	28,190 (24)	43,500 (20)	1,603 (24)	1,150 (21)	3,795 (23)	4,531 (21)	4,762 (22)	8,066 (20)	3,686 (21)	8,066 (20)	1,178 (20)	3,909 (20)	127 (18)	570 (19)
**Deprivation (IMD 2010)**														
SIMD 1 (most deprived)	29,992 (26)	50,116 (23)	1,701 (25)	1,194 (21)	4,209 (26)	4,817 (22)	5,920 (27)	9,229 (23)	5,081 (29)	10,575 (25)	5,081 (29)	5,889 (30)	273 (39)	996 (34)
SIMD 2	27,668 (24)	43,388 (22)	1,589 (24)	1,199 (22)	3,847 (23)	4,830 (22)	5,079 (23)	8,869 (22)	4,239 (25)	9,471 (23)	4,239 (25)	4,729 (24)	189 (27)	699 (24)
SIMD 3	22,473 (19)	43,892 (20)	1,238 (19)	1,131 (20)	3,109 (19)	4,280 (19)	4,148 (19)	7,936 (20)	3,212 (19)	8,154 (19)	3,212 (19)	3,669 (18)	107 (15)	568 (19)
SIMD 4	19,707 (17)	39,733 (18)	1,093 (16)	1,045 (19)	2,761 (17)	4,046 (18)	3,670 (17)	7,530 (19)	2,626 (15)	7,290 (17)	2,626 (15)	3,137 (16)	84 (12)	403 (14)
SIMD 5 (least deprived)	15,178 (13)	32,691 (15)	1,034 (16)	998 (18)	2,477 (15)	3,991 (18)	2,918 (13)	6,721 (16)	2,108 (12)	6,210 (15)	2,108 (12)	2,437 (12)	43 (6.2)	272 (9.2)
Unknown	324 (0.3)	730 (0.3)	16 (0.2)	11 (0.2)	16 (0.2)	63 (0.3)	36 (0.2)	127 (0.3)	43 (0.2)	137 (0.3)	43 (0.2)	74 (0.4)	1 (0.1)	6 (0.2)
**Duration of follow-up, years ***	5.6 ± 3.1	6.1 ± 3.1	5.2 ± 3.1	6.1 ± 9.2	5.5 ± 3.1	5.9 ± 3.2	5.7 ± 3.1	5.7 ± 3.1	5.7 ± 3.1	5.6 ± 3.1	5.9 ± 3.0	5.5 ± 3.0	5.9 ± 3.0	5.3 ± 2.9
**Duration of follow-up, years †**	5.9 (2.7-9.2)	6.8 (3.3-9.2)	5.0 (2.3-8.9)	7.1 (3.1-9.2)	5.5 (2.6-9.2)	6.4 (2.9-9.2)	5.9 (2.8-9.2)	6.0 (2.8-9.2)	6.0 (2.9-9.2)	5.8 (2.8-9.2)	6.3 (3.1-9.2)	5.5 (2.7-8.8)	6.2 (3.2-9.2)	5.3 (2.7-8.3)
**Risk Factors**														
**Current Smoker**	21,946 (19)	45,734 (21)	0	0	1,303 (7.9)	1,379 (6.2)	3,439 (16)	5,116 (12)	4,827 (28)	9,740 (23)	3,589 (60)	10,673 (54)	697 (100)	2,944 (100)
unknown (%)	5,569 (4.8)	13,741 (6.3)	-	-	-	-	-	-	-	-	-	-	-	-
**BMI ***	30.6 ± 5.8	32.1 ± 6.5	29.7 ± 5.5	30.8 ± 6.3	30.1 ± 5.7	31.4 ± 6.6	30.8 ± 5.8	31.9 ± 6.5	31.5 ± 5.8	32.5 ± 6.3	32.1 ± 6.0	33.0 ± 6.4	32.5 ± 5.9	33.2 ± 6.2
missing (%)	22,553 (20)	47,256 (22)	901 (14)	647 (12)	2015 (12)	2,986 (14)	2,741 (13)	5,864 (15)	2,199 (13)	6,461 (15)	890 (15)	3,221 (16)	98 (14)	496 (17)
**Total cholesterol ***	4.4 ± 1.1	5.0 ± 1.2	3.4 ± 0.4	3.4 ± 0.4	3.8 ± 0.8	4.3 ± 0.9	4.4 ± 1.0	4.9 ± 1.1	4.9 ± 1.0	5.4 ± 1.2	5.2 ± 1.1	5.7 ± 1.2	5.4 ± 1.2	5.9 ± 1.3
missing (%)	4,101 (3.5)	12,972 (6.0)	-	-	-	-	-	-	-	-	-	-	-	-
**HbA1c (mmol/mol) ***	58 ± 18	63 ± 21	46 ± 5.0	46 ± 5.0	53 ± 14	52 ± 14	58 ± 17	58 ± 18	66 ± 19	69 ± 22	72 ± 20	76 ± 22	74 ± 19	78 ± 20
missing (%)	4,931 (4.2)	13,057 (6.1)	-	-	-	-	-	-	-	-	-	-	-	-
**Blood pressure ***														

sBP	135 ± 18	138 ± 17	130 ± 15	132 ± 13	132 ± 16	133 ± 14	134 ± 17	135 ± 15	137 ± 18	138 ± 17	144 ± 20	146 ± 19	155 ± 16	157 ± 14
dBP	74 ± 10	80 ± 10	70 ± 8.0	74 ± 8.0	72 ± 9.0	76 ± 8.0	74 ± 9.0	78 ± 9.0	76 ± 10	81 ± 10	82 ± 11	86 ± 11	90 ± 7.0	92 ± 8.1
missing (%)	2,702 (2.3)	9,653 (4.4)	-	-	-	-	-	-	-	-	-	-	-	-
**Drug Prescriptions**														
**Diabetes**														
Any oral hyoglycaemic agent, %	48	35	40	42	45	36	47	34	50	32	52	31	47	27
Metformin, %	29	25	23	29	26	26	28	24	31	24	33	23	33	21
Sulphonylurea, %	22	13	19	16	20	14	21	12	22	11	22	10	18	8
Insulin, %	11	5.7	5.9	5.4	11	5.6	12	6.3	15	6.1	15	5.2	12	3.7
Other, %	6.3	5.0	4.9	5.7	5.5	5.1	6.0	4.6	6.8	4.4	7.6	4.2	7.5	3.0
**Antihypertensive agent**														
Any, %	81	51	87	68	85	61	84	55	82	48	79	43	80	42
Alpha-blocker, %	7.0	3.7	9.1	7.4	8.1	5.2	6.9	3.9	6.3	2.9	6.0	2.3	7.6	2.0
Angiotensin-2 receptor blocker, %	14	8.5	16	13	15	11	14	9.8	14	7.8	12	6.6	13	6.1
ACE inhibitor, %	46	26	50	37	48	30	47	28	47	25	46	23	48	23
Beta-blocker, %	44	17	51	24	47	20	47	19	45	16	42	14	41	12
Calcium channel blocker, %	34	18	39	29	37	24	35	20	34	17	33	15	33	15
Diuretic: thiazide, potassium sparing or loop, %	22	20	22	28	22	25	22	22	22	19	22	16	25	14
**Lipid lowering therapy**														
Any, %	72	40	84	67	78	50	74	42	72	36	71	31	71	31
**Antiplatelets**														
Any, %	64	22	71	39	68	29	66	23	64	18	62	16	51	14

Data presented as n(%), mean±SD * or median (25_{th}-75_{th} percentile) †, where indicated

Supplemental Table V. Age-standardised rates for cardiovascular events (fatal or non-fatal CVD event or heart failure hospitalisation) and mortality in people with type 2 diabetes and controls

	Database	Controls Rate/100 py (95% CI)	All T2D Rate/100 py (95% CI)	T2D with complete data on all 5 risk factors						
				Rate/100 py (95% CI)	*No. of risk factors above threshold; Rate/100 py (95% CI)*					
					**0**	**1**	**2**	**3**	**4**	**5**
N	CPRD	378,938	101,749	73,096	4,281	14,124	22,292	21,447	9,544	1,408
	SCI-Diabetes	-	330,892	201,653	12,249	38,474	62,183	59,146	25,960	3,641
**CVD events**										
**Total** (fatal/non-fatal CHD or stroke or heart failure hospitalisation)	CPRD	2.58 (2.55-2.60)	4.79 (4.71-4.88)	4.22 (4.14-4.31)	5.55 (5.06-6.04)	4.21 (4.01-4.41)	4.06 (3.83-4.28)	3.94 (3.81-4.08)	4.02 (3.77-4.28)	4.41 (3.70-5.12)
	SCI-Diabetes	-	3.39 (3.36-3.41)	5.19 (5.16-5.23)	5.84 (5.68-6.01)	5.08 (5.01-5.16)	4.86 (4.80-4.92)	5.12 (5.05-5.18)	5.56 (5.41-5.71)	5.56 (4.92-6.21)
**Non-fatal CHD**	CPRD	1.91 (1.89-1.93)	3.75 (3.68-3.81)	3.38 (3.31-3.45)	4.57 (4.23-4.92)	3.49 (3.30-3.68)	3.13 (3.02-3.24)	3.10 (2.99-3.22)	3.13 (2.93-3.33)	3.58 (2.91-4.24)
	SCI-Diabetes	-	3.44 (3.42-3.46)	3.60 (3.57-3.63)	4.14 (4.01-4.28)	3.61 (3.55-3.68)	3.36 (3.31-3.41)	3.52 (3.47-3.57)	3.66 (3.56-3.76)	3.71 (3.25-4.16)
**Non-fatal Stroke**	CPRD	0.48 (0.47-0.49)	0.70 (0.66-0.75)	0.58 (0.54-0.61)	0.46 (0.39-0.53)	0.48 (0.43-0.53)	0.61 (0.47-0.75)	0.59 (0.54-0.64)	0.61 (0.52-0.69)	0.93 (0.64-1.21)
	SCI-Diabetes	-	0.63 (0.62-0.63)	0.62 (0.61-0.64)	0.64 (0.59-0.70)	0.52 (0.50-0.54)	0.57 (0.55-0.59)	0.64 (0.62-0.66)	0.79 (0.74-0.85)	1.07 (0.82-1.32)
**Heart failure hospitalisation**	CPRD	0.71 (0.70-0.72)	1.36 (1.31-1.41)	1.18 (1.13-1.23)	1.48 (1.13-1.84)	1.12 (1.05-1.18)	1.14 (1.00-1.28)	1.14 (1.07-1.22)	1.19 (1.02-1.36)	1.29 (0.80-1.78)
	SCI-Diabetes	-	1.15 (1.14-1.16)	1.17 (1.16-1.19)	1.25 (1.18-1.32)	1.05 (1.02-1.08)	1.11 (1.08-1.14)	1.24 (1.21-1.28)	1.29 (1.22-1.37)	1.19 (0.91-1.47)
**Mortality**										
**Total** (CHD or stroke)	CPRD	0.33 (0.32-0.34)	0.45 (0.43-0.47)	0.37 (0.35-0.39)	0.47 (0.39-0.55)	0.36 (0.32-0.39)	0.34 (0.31-0.37)	0.37 (0.33-0.41)	0.41 (0.33-0.48)	0.39 (0.20-0.58)
	SCI-Diabetes	-	1.14 (1.13-1.15)	1.09 (1.07-1.11)	1.06 (1.00-1.12)	1.03 (1.00-1.06)	1.01 (0.98-1.04)	1.12 (1.08-1.15)	1.34 (1.24-1.43)	1.30 (0.99-1.63)
**CHD**	CPRD	0.18 (0.17-0.19)	0.28 (0.26-0.29)	0.23 (0.22-0.25)	0.30 (0.24-0.36)	0.21 (0.19-0.24)	0.22 (0.19-0.24)	0.23 (0.20-0.26)	0.25 (0.20-0.30)	0.27 (0.10-0.43)
	SCI-Diabetes	-	0.67 (0.66-0.68)	0.66 (0.65-0.68)	0.66 (0.61-0.71)	0.62 (0.60-0.64)	0.62 (0.60-0.64)	0.67 (0.65-0.70)	0.80 (0.74-0.87)	0.59 (0.49-0.70)
**Stroke**	CPRD	0.09 (0.08-0.10)	0.10 (0.09-0.11)	0.08 (0.07-0.09)	0.10 (0.07-0.14)	0.09 (0.07-0.11)	0.07 (0.06-0.08)	0.08 (0.06-0.10)	0.09 (0.05-0.14)	0.10 (0.01-0.19)
	SCI-Diabetes	-	0.21 (0.20-0.21)	0.19 (0.18-0.19)	0.17 (0.15-0.19)	0.17 (0.16-0.18)	0.17 (0.16-0.18)	0.20 (0.19-0.22)	0.23 (0.19-0.28)	0.32 (0.11-0.54)

Sub-optimal risk factor control thresholds: current smoker, total cholesterol>4 mmol/L, triglycerides>1.7 mmol/L, HbA1c≥7.0% (53mmol/mol), systolic blood pressure>140mmHg or >130mmHg in the presence of kidney or cerebrovascular damage

Supplemental Table VI. Age-standardised rates for cardiovascular events (fatal or non-fatal CVD event or heart failure hospitalisation) and mortality in people with type 2 diabetes and controls, stratified by the presence of cardio-renal disease

	Database	Prevalent cardio-renal disease †			No presence of cardio-renal disease †		
		Controls	All T2D	T2D with complete data on all 5 risk factors	Controls	All T2D	T2D with complete data on all 5 risk factors
		Rate/100 py (95% CI)	Rate/100 py (95% CI)	Rate/100 py (95% CI)	Rate/100 py (95% CI)	Rate/100 py (95% CI)	Rate/100 py (95% CI)
**N (%)**	CPRD	76,946 (20)	28,933 (28)	20,187 (28)	301,992 (80)	72,816 (72)	52,909 (72)
	SCI-Diabetes	-	115,342 (35)		-	215,550 (65)	
**CVD events**							
**Total** (fatal/non-fatal CHD or stroke or heart failure hospitalisation)	CPRD	8.27 (7.94-8.59)	16.1 (13.8 -18.5)	16.0 (8.58-23.5)	1.64 (1.62-1.67)	2.87 (2.79-2.95)	2.48 (2.40-2.56)
	SCI-Diabetes	-	13.8 (13.6-14.0)	14.2 (13.9-14.4)	-	2.61 (2.59-2.64)	2.63 (2.59-2.66)
**Non-fatal CHD**	CPRD	7.26 (6.98-7.54)	13.7 (12.2 -15.2)	11.2 (10.4 -12.0)	1.02 (1.01-1.04)	1.93 (1.88-1.98)	1.74 (1.68-1.80)
	SCI-Diabetes	-	10.8 (10.6-10.9)	11.3 (11.1-11.6)	-	1.37 (1.36-1.39)	1.39 (1.37-1.42)
**Non-fatal Stroke**	CPRD	0.90 (0.74-1.07)	1.02 (0.91-1.13)	0.91 (0.76-1.05)	0.41 (0.39-0.42)	0.58 (0.54-0.63)	0.46 (0.42-0.50)
	SCI-Diabetes	-	1.02 (0.90-1.07)	0.96 (0.92-1.00)	-	0.46 (0.45-0.47)	0.46 (0.44-0.47)
**Heart failure hospitalisation**	CPRD	1.50 (1.42-1.58)	3.62 (1.79-5.44)	5.87 (1.53-13.28)	0.49 (0.48-0.50)	0.89 (0.85-0.94)	0.74 (0.70-0.79)
	SCI-Diabetes	-	2.07 (2.02-2.12)	2.08 (2.02-2.15)	-	0.62 (0.61-0.64)	0.64 (0.62-0.66)
**Mortality**							
**Total** (CHD or stroke)	CPRD	0.66 (0.60-0.72)	0.92 (0.64-1.19)	0.65 (0.59-0.70)	0.23 (0.22-0.23)	0.28 (0.26-0.30)	0.23 (0.21-0.25)
	SCI-Diabetes	-	1.74 (1.70-1.77)	1.67 (1.62-1.72)	-	0.69 (0.68-0.70)	0.65 (0.63-0.68)
**CHD**	CPRD	0.38 (0.36-0.41)	0.54 (0.48-0.60)	0.45 (0.40-0.49)	0.11 (0.11-0.12)	0.16 (0.15-0.18)	0.14 (0.12-0.15)
	SCI-Diabetes	-	1.10 (1.08-1.13)	1.09 (1.06-1.12)	-	0.36 (0.35-0.37)	0.34 (0.33-0.36)
**Stroke**	CPRD	0.15 (0.13-0.17)	0.14 (0.12-0.16)	0.11 (0.10-0.13)	0.07 (0.06-0.07)	0.07 (0.06-0.08)	0.05 (0.04-0.06)
	SCI-Diabetes	-	0.27 (0.24-0.29)	0.26 (0.23-0.30)	-	0.15 (0.14-0.15)	0.14 (0.13-0.15)

† Cardio-renal disease defined as: prior history of acute MI, stroke, CHD and/or renal impairment

Supplemental Figures

Supplemental Figure I. Structure of hospital in-patient data in England and Scotland

Patient 1

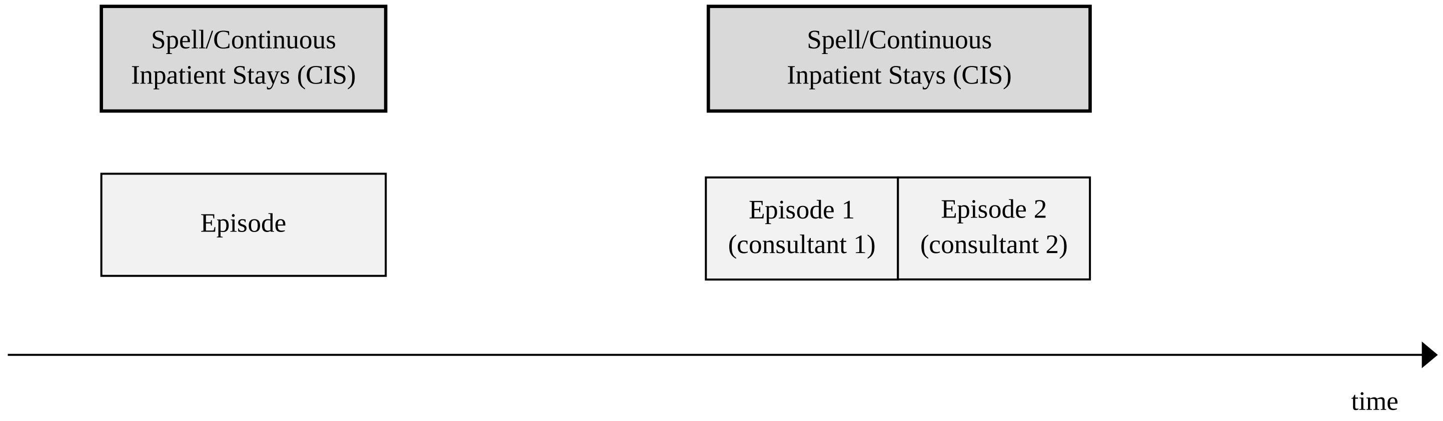

Episode: This is defined as a single period of care under one consultant.[39]

Spell (England)/Continuous Inpatient Stays (Scotland): This is defined as the total period of inpatient hospital stay. A spell or CIS can contain one or more episodes; however, the vast majority of spells/CIS contain only one episode. Each patient can have one or more hospital spells/CIS. If the patient is transferred to another hospital, dies or is discharged, the spell/CIS and the episode end.[39]

In the CPRD dataset, CVD events were captured across all diagnosis fields from HES admitted patient care, including; diagnoses recorded in an episode, diagnoses recorded within a hospitalisation (spell) and primary diagnoses across a hospitalisation (first diagnosis recorded during each episode of care in a spell). In the SCI-diabetes cohort, CVD events recorded in hospital were captured across Continuous inpatient Stays (CIS) and episodes of care.

Supplemental Figure II. Meta-analysis of multivariable-adjusted relative hazards for CHD, stroke and HF hospitalisation according to number of risk factors above thresholds in people with type 2 diabetes from CPRD and SCI-Diabetes compared to people with optimal risk factor control

CVD Events

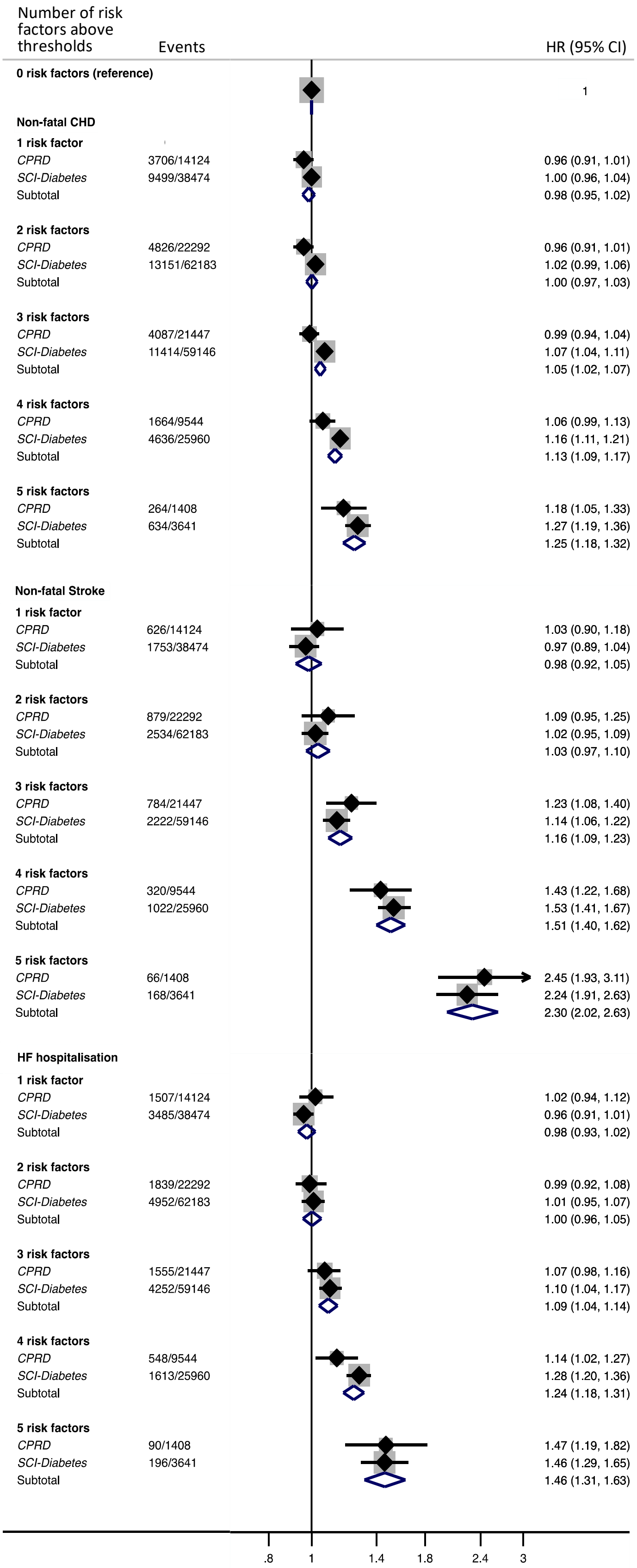

CVD mortality

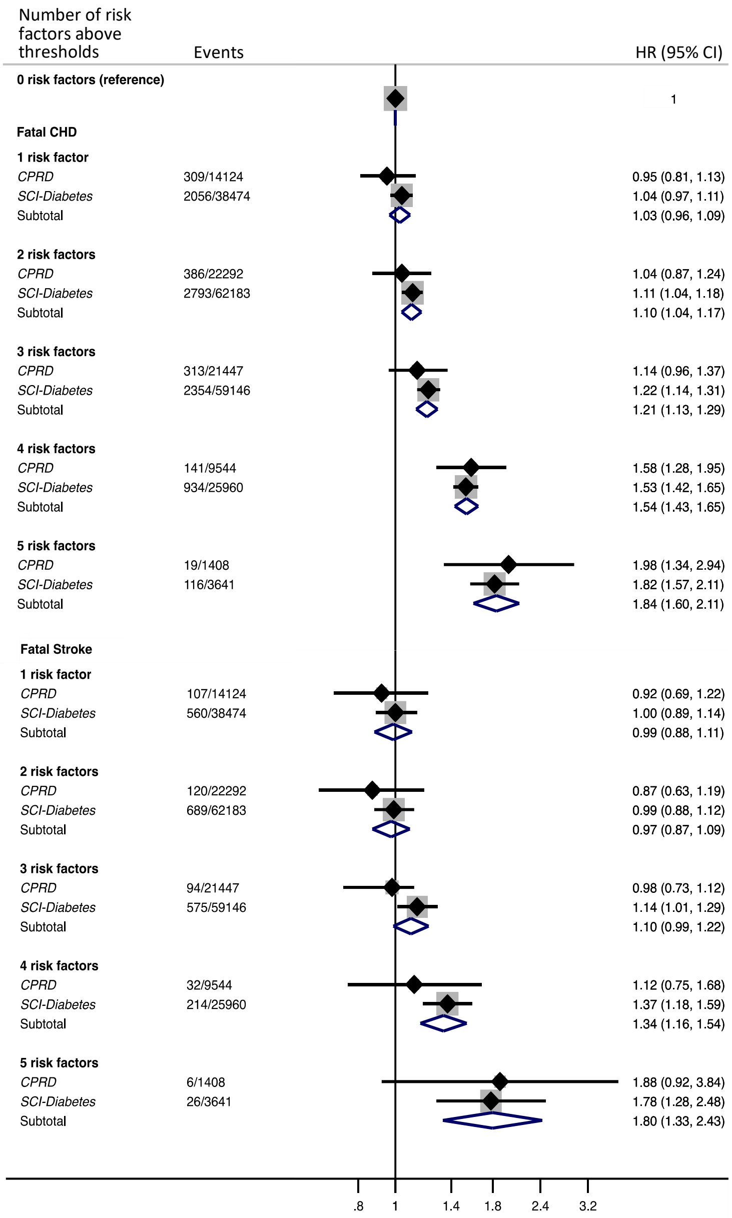

Adjusted for age, sex, deprivation, ethnicity, diabetes duration and history of CVD. Confidence intervals Bonferroni adjusted to account for multiplicity. Hazard ratios are pooled from all 5 imputed data sets. Number of events and population represent the mean in the 5 data sets.

Supplemental Figure III. Meta-analysis of multivariable-adjusted relative hazards for CHD, stroke and HF hospitalisation according to number of risk factors above thresholds in people with type 2 diabetes from CPRD and SCI-Diabetes compared to people with optimal risk factor control, stratified by the presence of cardio-renal disease

A: CVD events

T2D with prevalent cardio-renal disease

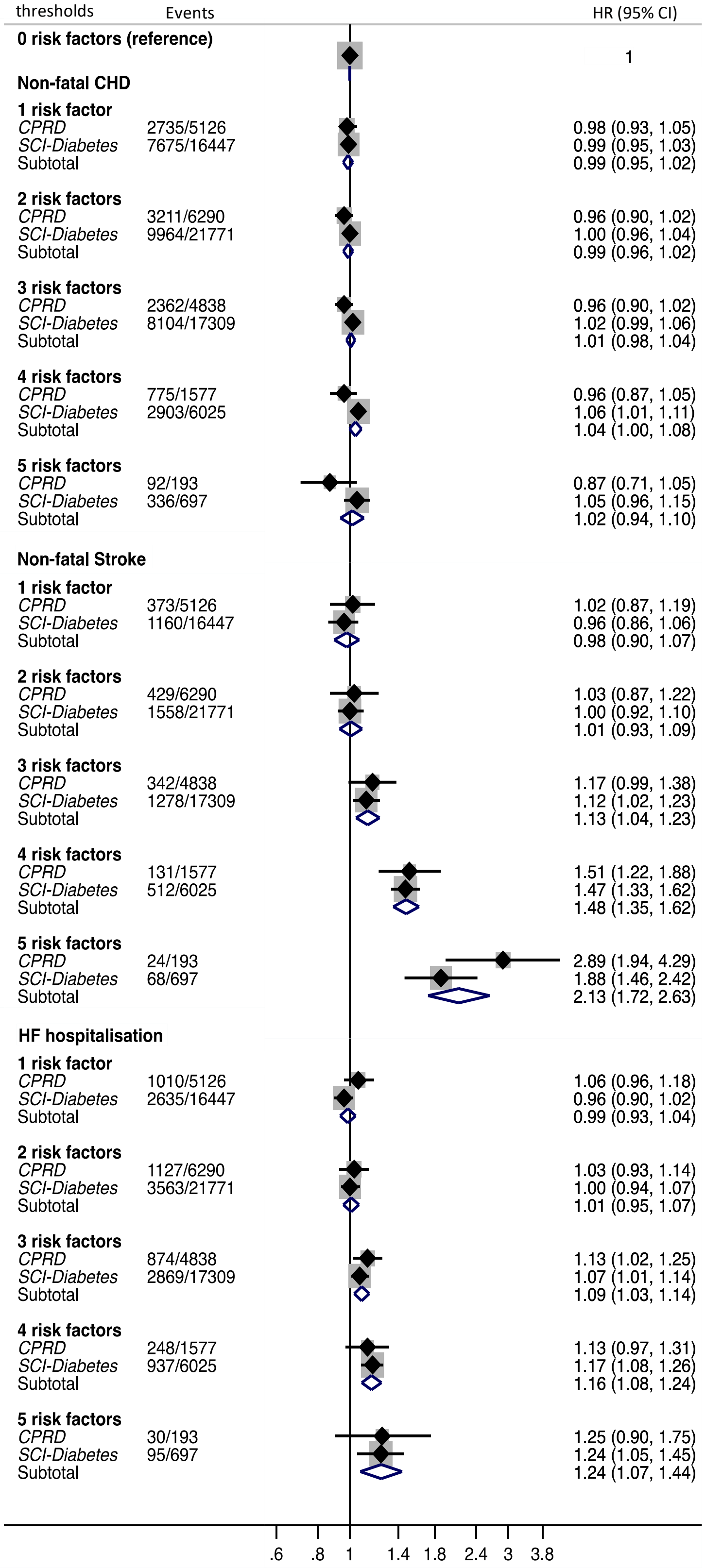

Number of risk factors above thresholds	Events	HR (95% CI)
**0 risk factors (reference)**		1
**Non-fatal CHD**		
**1 risk factor**		
CPRD	2735/5126	0.98 (0.93, 1.05)
SCI-Diabetes	7675/16447	0.99 (0.95, 1.03)
Subtotal		0.99 (0.95, 1.02)
**2 risk factors**		
CPRD	3211/6290	0.96 (0.90, 1.02)
SCI-Diabetes	9964/21771	1.00 (0.96, 1.04)
Subtotal		0.99 (0.96, 1.02)
**3 risk factors**		
CPRD	2362/4838	0.96 (0.90, 1.02)
SCI-Diabetes	8104/17309	1.02 (0.99, 1.06)
Subtotal		1.01 (0.98, 1.04)
**4 risk factors**		
CPRD	775/1577	0.96 (0.87, 1.05)
SCI-Diabetes	2903/6025	1.06 (1.01, 1.11)
Subtotal		1.04 (1.00, 1.08)
**5 risk factors**		
CPRD	92/193	0.87 (0.71, 1.05)
SCI-Diabetes	336/697	1.05 (0.96, 1.15)
Subtotal		1.02 (0.94, 1.10)
**Non-fatal Stroke**		
**1 risk factor**		
CPRD	373/5126	1.02 (0.87, 1.19)
SCI-Diabetes	1160/16447	0.96 (0.86, 1.06)
Subtotal		0.98 (0.90, 1.07)
**2 risk factors**		
CPRD	429/6290	1.03 (0.87, 1.22)
SCI-Diabetes	1558/21771	1.00 (0.92, 1.10)
Subtotal		1.01 (0.93, 1.09)
**3 risk factors**		
CPRD	342/4838	1.17 (0.99, 1.38)
SCI-Diabetes	1278/17309	1.12 (1.02, 1.23)
Subtotal		1.13 (1.04, 1.23)
**4 risk factors**		
CPRD	131/1577	1.51 (1.22, 1.88)
SCI-Diabetes	512/6025	1.47 (1.33, 1.62)
Subtotal		1.48 (1.35, 1.62)
**5 risk factors**		
CPRD	24/193	2.89 (1.94, 4.29)
SCI-Diabetes	68/697	1.88 (1.46, 2.42)
Subtotal		2.13 (1.72, 2.63)
**HF hospitalisation**		
**1 risk factor**		
CPRD	1010/5126	1.06 (0.96, 1.18)
SCI-Diabetes	2635/16447	0.96 (0.90, 1.02)
Subtotal		0.99 (0.93, 1.04)
**2 risk factors**		
CPRD	1127/6290	1.03 (0.93, 1.14)
SCI-Diabetes	3563/21771	1.00 (0.94, 1.07)
Subtotal		1.01 (0.95, 1.07)
**3 risk factors**		
CPRD	874/4838	1.13 (1.02, 1.25)
SCI-Diabetes	2869/17309	1.07 (1.01, 1.14)
Subtotal		1.09 (1.03, 1.14)
**4 risk factors**		
CPRD	248/1577	1.13 (0.97, 1.31)
SCI-Diabetes	937/6025	1.17 (1.08, 1.26)
Subtotal		1.16 (1.08, 1.24)
**5 risk factors**		
CPRD	30/193	1.25 (0.90, 1.75)
SCI-Diabetes	95/697	1.24 (1.05, 1.45)
Subtotal		1.24 (1.07, 1.44)

T2D without cardio-renal disease

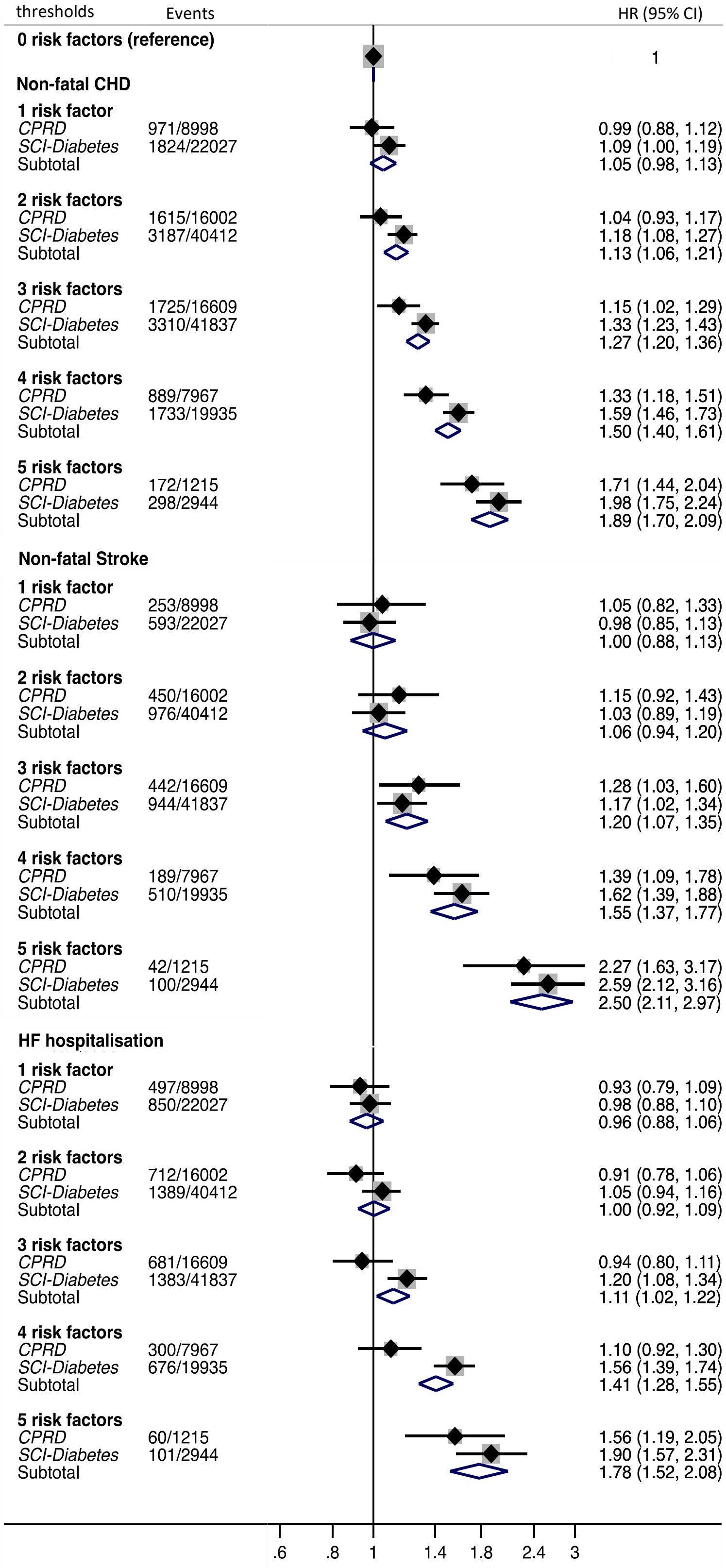

Number of risk factors above thresholds	Events	HR (95% CI)
**0 risk factors (reference)**		1
**Non-fatal CHD**		
**1 risk factor**		
CPRD	971/8998	0.99 (0.88, 1.12)
SCI-Diabetes	1824/22027	1.09 (1.00, 1.19)
Subtotal		1.05 (0.98, 1.13)
**2 risk factors**		
CPRD	1615/16002	1.04 (0.93, 1.17)
SCI-Diabetes	3187/40412	1.18 (1.08, 1.27)
Subtotal		1.13 (1.06, 1.21)
**3 risk factors**		
CPRD	1725/16609	1.15 (1.02, 1.29)
SCI-Diabetes	3310/41837	1.33 (1.23, 1.43)
Subtotal		1.27 (1.20, 1.36)
**4 risk factors**		
CPRD	889/7967	1.33 (1.18, 1.51)
SCI-Diabetes	1733/19935	1.59 (1.46, 1.73)
Subtotal		1.50 (1.40, 1.61)
**5 risk factors**		
CPRD	172/1215	1.71 (1.44, 2.04)
SCI-Diabetes	298/2944	1.98 (1.75, 2.24)
Subtotal		1.89 (1.70, 2.09)
**Non-fatal Stroke**		
**1 risk factor**		
CPRD	253/8998	1.05 (0.82, 1.33)
SCI-Diabetes	593/22027	0.98 (0.85, 1.13)
Subtotal		1.00 (0.88, 1.13)
**2 risk factors**		
CPRD	450/16002	1.15 (0.92, 1.43)
SCI-Diabetes	976/40412	1.03 (0.89, 1.19)
Subtotal		1.06 (0.94, 1.20)
**3 risk factors**		
CPRD	442/16609	1.28 (1.03, 1.60)
SCI-Diabetes	944/41837	1.17 (1.02, 1.34)
Subtotal		1.20 (1.07, 1.35)
**4 risk factors**		
CPRD	189/7967	1.39 (1.09, 1.78)
SCI-Diabetes	510/19935	1.62 (1.39, 1.88)
Subtotal		1.55 (1.37, 1.77)
**5 risk factors**		
CPRD	42/1215	2.27 (1.63, 3.17)
SCI-Diabetes	100/2944	2.59 (2.12, 3.16)
Subtotal		2.50 (2.11, 2.97)
**HF hospitalisation**		
**1 risk factor**		
CPRD	497/8998	0.93 (0.79, 1.09)
SCI-Diabetes	850/22027	0.98 (0.88, 1.10)
Subtotal		0.96 (0.88, 1.06)
**2 risk factors**		
CPRD	712/16002	0.91 (0.78, 1.06)
SCI-Diabetes	1389/40412	1.05 (0.94, 1.16)
Subtotal		1.00 (0.92, 1.09)
**3 risk factors**		
CPRD	681/16609	0.94 (0.80, 1.11)
SCI-Diabetes	1383/41837	1.20 (1.08, 1.34)
Subtotal		1.11 (1.02, 1.22)
**4 risk factors**		
CPRD	300/7967	1.10 (0.92, 1.30)
SCI-Diabetes	676/19935	1.56 (1.39, 1.74)
Subtotal		1.41 (1.28, 1.55)
**5 risk factors**		
CPRD	60/1215	1.56 (1.19, 2.05)
SCI-Diabetes	101/2944	1.90 (1.57, 2.31)
Subtotal		1.78 (1.52, 2.08)

Cardio-renal disease defined as: prior history of acute MI, stroke, CHD and/or renal impairment. Adjusted for age, sex, deprivation, ethnicity and diabetes duration. Confidence intervals Bonferroni adjusted to account for multiplicity. Hazard ratios are pooled from all 5 imputed data sets. Number of events and population represent the mean in the 5 data sets.

B: CVD mortality

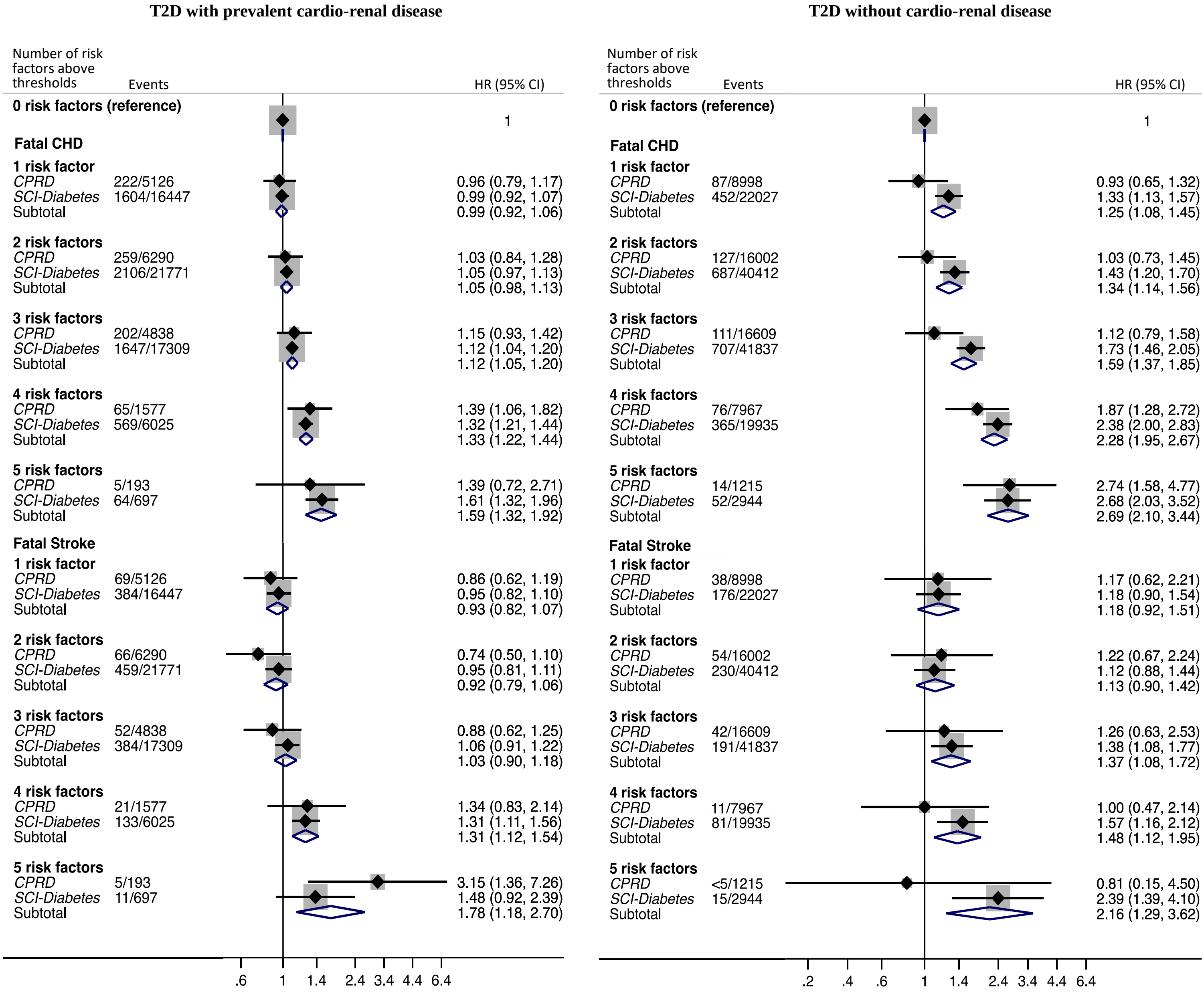

Cardio-renal disease defined as: prior history of acute MI, stroke, CHD and/or renal impairment. Adjusted for age, sex, deprivation, ethnicity and diabetes duration. Confidence intervals Bonferroni adjusted to account for multiplicity. Hazard ratios are pooled from all 5 imputed data sets. Number of events and population represent the mean in the 5 data sets.

Supplemental Reference

39. Methodology to create provider and CIP spells from HES APC data . Health & Social Care Information Centre. 2014. Available from: https://digital.nhs.uk/binaries/content/documents/corporate-website/publication-system/ci-hub/compendium-indicators/compendium-indicators/publicationsystem%3Acilandingasset[3]/publicationsystem%3AAttachments[12]/publicationsystem%3AattachmentResource
